# Dawn-to-dusk dry fasting induces anti-atherosclerotic, anti-inflammatory, and anti-tumorigenic proteome in peripheral blood mononuclear cells in subjects with metabolic syndrome

**DOI:** 10.1016/j.metop.2022.100214

**Published:** 2022-11-01

**Authors:** Ayse L. Mindikoglu, Jihwan Park, Antone R. Opekun, Mustafa M. Abdulsada, Zoe R. Wilhelm, Prasun K. Jalal, Sridevi Devaraj, Sung Yun Jung

**Affiliations:** a Margaret M. and Albert B. Alkek Department of Medicine, Section of Gastroenterology and Hepatology, Baylor College of Medicine, Houston, TX, USA; b Michael E. DeBakey Department of Surgery, Division of Abdominal Transplantation, Baylor College of Medicine, Houston, TX, USA; c Department of Molecular & Cellular Biology, Baylor College of Medicine, Houston, TX, USA; d Department of Pediatrics, Division of Gastroenterology, Nutrition and Hepatology, Baylor College of Medicine, Houston, TX, USA; e Clinical Chemistry and Point of Care Technology, Texas Children's Hospital, Department of Pathology and Immunology, Baylor College of Medicine, Houston, TX, USA

**Keywords:** Dry fasting, Intermittent fasting, Dawn-to-dusk dry fasting, Metabolic syndrome, Proteomics, Proteome, Peripheral blood mononuclear cell, PBMC, Apolipoprotein B, APOB, Diurnal fasting, Daytime fasting, Obesity, Non-alcoholic fatty liver disease, Fatty liver, NAFLD, Ramadan fasting

## Abstract

**Background:**

Metabolic syndrome characterized by abdominal obesity, high blood pressure, elevated fasting glucose and triglyceride levels and low high-density lipoprotein cholesterol level is associated with pro-inflammatory state, increased risk for atherosclerosis, and multiple cancers. Our previous results on subjects with metabolic syndrome showed that 4-week dawn-to-dusk (sunset) dry fasting resulted in significant changes in the serum proteome and improvement in several metabolic risk factors. Peripheral blood mononuclear cells (PBMC) proteomics is a powerful tool that can provide mechanistic insights into how dawn-to-dusk dry fasting affects protein expression in metabolic pathways at cellular level. In this study, we determined whether dawn-to-dusk dry fasting would induce favorable changes in PBMC proteome in subjects with metabolic syndrome, similar to the changes induced by dawn-to-dusk dry fasting in the same subjects' serum proteome.

**Methods:**

We conducted a prospective study on subjects with metabolic syndrome and collected blood specimens before 4-week dawn-to-dusk dry fasting, at the end of 4-week dawn-to-dusk dry fasting, and one week after 4-week dawn-to-dusk dry fasting. We performed untargeted proteomics using nano ultra-high performance liquid chromatography-tandem mass spectrometry to assess the impact of 4-week dawn-to-dusk dry fasting on PBMC proteome.

**Results:**

There were 14 subjects with metabolic syndrome with a mean age of 59 who fasted from dawn to dusk (strict dry fasting without any liquid or food intake) for more than 14 h daily for 29 days. The quantitative proteome analysis showed that apolipoprotein B (APOB) gene protein products (GP) levels were downregulated and had the most statistical significance of the observed difference at the end of 4-week dawn-to-dusk dry fasting (P = 0.008) and one week after 4-week dawn-to-dusk dry fasting (P = 0.0004) compared with the levels before 4-week dawn-to-dusk dry fasting. The comparison between GP levels before and at the end of 4-week dawn-to-dusk dry fasting showed an alteration in the expression of genes associated with lipid and atherosclerosis pathway (P = 6.014e-4) and C-type lectin receptor signaling pathway (P = 1.064e-5). The genes that were differentially expressed in the lipid and atherosclerosis pathway were APOB (P = 0.008), CD36 (P = 0.040), CALM1, CALM2, CALM3 (P = 0.015), and HSPA8 (P = 0.047). One of the differentially expressed genes in the C-type lectin receptor signaling pathway was lymphocyte-specific protein 1 (LSP1), which showed an average of 19-fold increase at the end of 4-week dawn-to-dusk dry fasting compared with the GP levels before fasting (P = 0.004). Several GPs associated with tumor-suppressor effect (TUBB4B, LSP1, ACTR3B) were upregulated, and GPs associated with tumor-promoter effect (CD36, CALM1, CALM2, CALM3, FLOT2, PPIF) were downregulated at the end of 4-week dawn-to-dusk dry fasting or one week after 4-week dawn-to-dusk dry fasting compared with the GP levels before 4-week dawn-to-dusk dry fasting.

**Conclusion:**

Based on our results, we conclude that in subjects with metabolic syndrome, 4-week dawn-to-dusk dry fasting induced anti-atherosclerotic, anti-inflammatory, and anti-tumorigenic PMBC proteome. Randomized, controlled clinical trials are needed to further investigate the effect of dawn-to-dusk dry fasting on subjects with chronic metabolic diseases and metabolic syndrome-induced cancers.

## Graphical abstract

**Figure:**
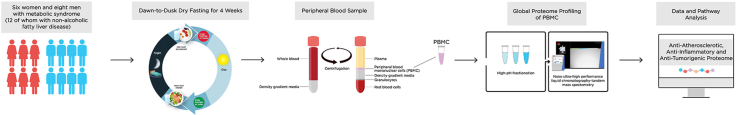

## 1 Introduction

According to World Health Organization, every year, nearly 3 million people die worldwide due to the complications of increased body mass index [1]. Eating patterns not aligned with the circadian clock rhythm (e.g., ad libitum eating, skipping breakfast, late-night eating) result in a disrupted circadian rhythm that can lead to obesity and metabolic syndrome [[2], [3], [4]]. Metabolic syndrome, which is a circadian rhythm disorder [5] is characterized by abdominal obesity, insulin resistance, elevated blood pressure, low blood high-density lipoprotein cholesterol and high blood triglyceride levels [6], and associated with a pro-inflammatory state, increased risk for atherosclerotic cardiovascular disease, and multiple cancers [[7], [8], [9], [10], [11]].

**Fig. 1:**
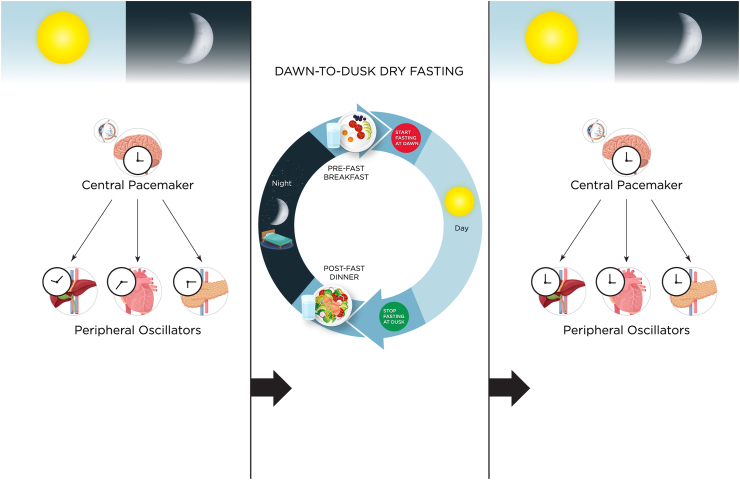
Dawn-to-dusk dry fasting to align and synchronize circadian clocks with dawn and dusk: Hypothetical reset of the circadian clock. Ad libitum eating and drinking, and mealtimes not aligned with dawn and dusk will result in misalignment of peripheral oscillators/clocks with the central pacemaker located in the suprachiasmatic nucleus of the hypothalamus (clocks showing different internal circadian time). Morning and evening circadian oscillators play a critical role in human metabolism and are entrained to dawn and dusk [[13], [14], [15]]. Therefore, dawn-to-dusk dry fasting with meals just before and after the fast for several consecutive days likely act as a robust zeitgeber by aligning meals with dawn and dusk (e.g., solar light-dark cycles). Aligning mealtimes with dawn and dusk will, in turn, synchronize the phase of peripheral oscillators with the phase of the central pacemaker in the suprachiasmatic nucleus (clocks showing the same internal circadian time after dawn-to-dusk dry fasting), normalize the circadian phase, and optimize the amplitude of the mRNA oscillations (thereby protein synthesis).

In Neurospora crassa, a model organism, the peak timing of clock-controlled gene transcription and expression was shown to be exclusive to dawn and dusk [12]. Similarly, in humans, morning and evening oscillators of the circadian clock are entrained to dawn and dusk, respectively, and play a critical role in metabolism [[13], [14], [15]]. The central clock is entrained primarily by solar light-dark cycles (e.g., scotoperiod/duration of night time) and functions as a zeitgeber (a time giver, rhythmic environmental cue) to all peripheral clocks [16,17]. An exception to the dominance of the central clock over peripheral clocks is the entrainment of peripheral clocks by consecutive rhythmic fasting and eating cycles that release the peripheral clocks from the control of the central clock [16]. In contrast to the central clock, which is only weakly entrained by mealtimes, the peripheral clocks are dominantly entrained by mealtimes [16]. Therefore, meals immediately before and after a dawn-to-dusk fast can act as a robust zeitgeber to peripheral clocks and align them with the central clock phase entrained to dawn and dusk (Fig. 1). When mealtimes are not aligned with dawn and dusk for several consecutive days, entrainment of peripheral clocks can result in circadian phase differences between central and peripheral clocks (Fig. 1). The result can be an altered amplitude of mRNA synthesis for clock proteins, which changes downstream protein synthesis and results in metabolic dysfunction, as shown in eating patterns not aligned with metabolic regulators [18,19]. Breakfast skippers and late-night eaters have disrupted circadian cortisol rhythm, likely secondary to the circadian phase difference between the scotoperiod and mealtimes [2,3]. Glucocorticoids (e.g., cortisol) prevent rapid circadian phase shifts in the peripheral clocks that can occur due to the uncoupling of the peripheral clocks from the central clock after several consecutive days of fasting/refeeding cycles [20]. Subjects who fasted from dawn to dusk had two cortisol acrophases (peaks) during dawn and dusk vs. only one acrophase in subjects who did not fast [21]. These findings suggest that when mealtimes are scheduled just before and after a dawn-to-dusk fast, the biphasic cortisol circadian rhythm during fasting synchronizes the phase of peripheral clocks to the phase of the central clock and prevents phase shifts between the central and peripheral clocks. This epiphenomenon may have profound effects because dawn-to-dusk dry fasting for consecutive days with meals just before and after fasting can synchronize central clock and peripheral clocks and enhance the amplitude of mRNA oscillations, affecting downstream protein synthesis at multiple metabolic pathways.

Fasting periods with mealtimes not at dawn and dusk provided no significant benefits for an intermittent fasting/time-restricted eating diet compared with controls. A 12-week randomized controlled trial with overweight and obese subjects found that fasting for 16 h from 8 p.m. until noon the next day, with the first meal at noon, resulted in no significant changes in weight or metabolic risk factors compared to eating three structured meals a day with snacks between meals [22]. This suggests that skipping breakfast and eating the first meal at noon (phase-delayed eating) may have reduced the positive effects of time-restricted eating in this study. Also, consuming non-caloric drinks or water during the fast could reduce the impact of the meal as the dominant cue for peripheral circadian oscillators.

We previously reported that dawn-to-dusk (sunset) dry fasting was associated with anti-cancer serum proteome response and upregulated several key regulatory proteins of tumor suppression, DNA repair, insulin signaling, and immune regulation in healthy subjects and subjects with metabolic syndrome [23,24]. These changes in serum proteome at the end of 4-week dawn-to-dusk dry fasting and one week after 4-week dawn-to-dusk dry fasting also coincided with the improvement in several metabolic risk factors in subjects with metabolic syndrome (e.g., body weight, waist circumference, blood pressure, insulin resistance) [23,24].

PBMC proteomics is a powerful tool that can provide mechanistic insights into the effect of dawn-to-dusk dry fasting on the proteome and affected metabolic pathways. This study aimed to determine whether 4-week dawn-to-dusk dry fasting would induce favorable changes in peripheral blood mononuclear cells (PBMC) proteome in subjects with metabolic syndrome, similar to the changes induced by 4-week dawn-to-dusk dry fasting in the same subjects’ serum proteome [24].

## 2 Methods

### 2.1 Study subjects

This study was approved by the Baylor College of Medicine Institutional Review Board (Protocol number H-31612). All study participants provided signed informed consent. The inclusion and exclusion criteria of this study were previously reported [24]. In brief, subjects 18 years or older and diagnosed with metabolic syndrome were included in the study [24]. Metabolic syndrome was diagnosed based on the criteria described by Grundy et al. [6] Subjects who could not provide informed consent and those who had a cardiovascular event during the last six months before enrollment, active alcohol/recreational substance use, active cancer, infection, or seizure disorder were excluded [24]. Female subjects who were pregnant or breastfeeding were also excluded [24].

### 2.2 Study procedures

Fasting blood samples were collected before 4-week dawn-to-dusk dry fasting, at the end of 4-week dawn-to-dusk dry fasting, and one week after 4-week dawn-to-dusk dry fasting.

### 2.3 Proteomic analysis of peripheral blood mononuclear cells (PBMC)

Nano ultra-high performance liquid chromatography-tandem mass spectrometry (nano UHPLC-MS/MS) was performed for quantitative analysis of PMBC samples. To isolate PBMC, we used BD Vacutainer® CPT™, a cell preparation tube containing an anticoagulant, and FICOLL™ Hypaque™ solution as mononuclear cell separation media from whole blood. The frozen PBMC aliquot was thawed on ice, and cell debris were recovered by insertion of the micro-pipet dip into to PBMC solution. The cell aggregate attached to the micro pipet tip was resuspended briefly in cold phosphate-buffered saline (PBS) and spun down at 500×*g* for 1 min. The washed cell pellet was resuspended with 10 sample volumes of 50 mM ammonium bicarbonate with 1 mM CaCl2. Cell suspensions were lysed by 30-sec sonication (25-amp, 30-sec pulse). The protein concentration was measured by a commercial Bradford assay kit (Pierce™ Coomassie Plus (Bradford) Assay Kit, cat#23236), and 25 μg of total protein was digested with 0.5 μg of trypsin/Lys-C mix (A40007, Thermo Scientific) for 12 h. The lysate was further digested with 0.5 μg of trypsin for 4 h. The peptide concentration was measured using Colorimetric Peptide Assay (Thermo Scientific 23275), and 10 μg of the peptide from each sample was enriched by an in-housed STAGE tip column with 2 mg of C18 beads (3 μm, Dr. Maisch GmbH, Germany) [25] and vacuum dried. Resuspended peptides were subjected to a nanoLC-1000 (Thermo Scientific) coupled with an Orbitrap Fusion mass spectrometer (Thermo Scientific) with an ESI source. The peptides were loaded onto an in-house Reprosil-Pur Basic C18 (1.9 μm, Dr. Maisch GmbH, Germany) trap column (2 cm length, 100 μm i.d.) and separated by a 5 cm column (150 μm i.d.) with a 75 min gradient of 2–28% of acetonitrile/0.1% formic acid at a flow rate of 800 nl/min. Obtained spectra were searched against the target-decoy Human RefSeq database (release 2020) in Proteome Discoverer 2.1 interface (PD 2.1, Thermo Fisher) with the Mascot algorithm (Mascot 2.4, Matrix Science). Dynamic modifications of the acetylation of N-terminus and oxidation of methionine were allowed. The precursor mass tolerance was confined within 20 ppm with fragment mass tolerance of 0.5 Da, and a maximum of two missed cleavages was allowed. Assigned peptides were filtered with a 1% false discovery rate using percolator validation based on q-value. Label-free proteomics data were assigned to gene ID and calculated with the intensity-based absolute quantification (iBAQ) algorithm for abundance by Grouper [26]. Gene protein product (GP) quantification was performed as previously described [23,24]. The GPs’ iBAQ was calculated by dividing the total area-under-curve (AUC) of peptides belonging to the GPs by the peptide capacity (the number of tryptic peptides) that results from a given GP. Here, the peptide capacity is calculated on the gene level, consistent with this gene-centric approach to proteomics. For the cases in which there are multiple isoforms per gene, the peptide capacity is the average number of singly miscut peptides across all the isoforms [23,24].

### 2.4 Statistical analysis

Excel application was used for statistical analysis of PBMC proteomics (Microsoft, Redmond, WA). To determine differentially expressed protein levels at the end of 4-week dawn-to-dusk dry fasting and one week after 4-week dawn-to-dusk dry fasting, paired two-tailed student's t-tests were performed using log converted intensity-based fraction of total (iFOT) value which is defined as iBAQ of the individual identified protein divided by the total iBAQ of all identified proteins within one experiment [23,24]. Differential gene expression was considered if the GP level showed greater than or equal to 1.5-fold (log2 fold greater than or equal to 0.585) average paired change and a P-value of <0.05 [23,24]. Volcano plot analysis was performed to display the GPs that had greater than or equal to 1.5-fold significant change at the end of 4-week dawn-to-dusk dry fasting and one week after 4-week dawn-to-dusk dry fasting [[23], [24]].

The pathways and biological processes were identified using Advaita Bio's iPathwayGuide [[27], [28], [29], [30]]. Protein levels that showed greater than or equal to 1.5-fold (log2 fold greater than or equal to 0.585) average paired change and a P-value of <0.05 at the end of 4-week dawn-to-dusk dry fasting and one week after 4-week dawn-to-dusk dry fasting were considered as significant.

SAS software, Version 9.4 TS Level 1M7 X64_10PRO platform (SAS Institute Inc., Cary, NC, USA) was used to perform statistical analyses [31]. Paired t-tests were used to determine statistically significant differences in the parameter levels at the end of 4-week dawn-to-dusk dry fasting and one week after 4-week dawn-to-dusk dry fasting.

Pearson's correlation coefficients were calculated to assess correlations between fold changes in the differentially expressed GPs and the components of metabolic syndrome, lipid panel, hepatic panel, and adiposity, oxidative stress, and inflammation biomarkers at the end of 4-week dawn-to-dusk dry fasting and one week after 4-week dawn-to-dusk dry fasting compared with the levels before 4-week dawn-to-dusk dry fasting. In these analyses, a two-tailed *P* value of < 0.05 was considered statistically significant.

## 3 Results

**Fig. 2:**
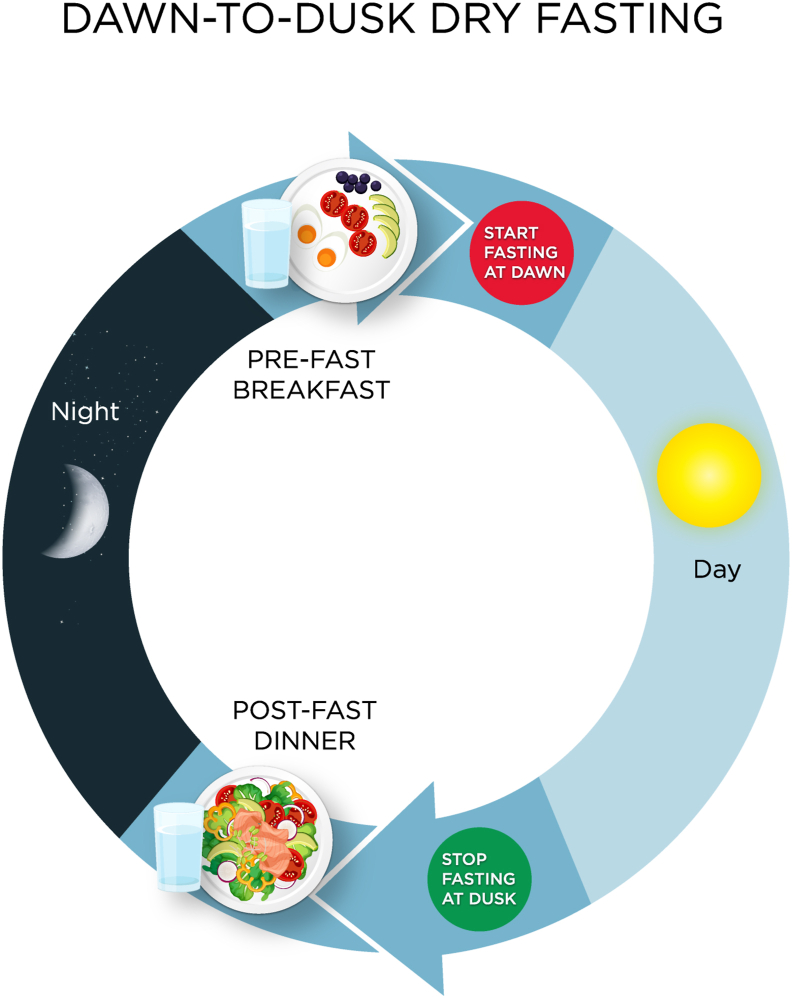
**Dawn-to-dusk dry fasting.** Subjects fasted from dawn to dusk without eating or drinking (nothing by mouth) for more than 14 h daily for 29 days. They had a pre-fast meal (breakfast) before they started fasting at dawn and a post-fast meal (dinner) after they stopped fasting at dusk (after sunset).

Fourteen subjects (8 men and 6 women, with a mean age of 59 years [SD = 16]) with metabolic syndrome, twelve of whom with nonalcoholic fatty liver disease, were enrolled in the study. Subjects fasted (strict dry fasting without food or drink intake) from dawn to dusk (sunset) for more than 14 h daily for 29 days during the month of Ramadan [32] in 2019 (Fig. 2). All subjects tolerated 4-week dawn-to-dusk dry fasting well without any side effects.

### 3.1 Changes in the gene protein products (GP) at the end of 4-week dawn-to-dusk dry fasting compared with GP levels before 4-week dawn-to-dusk dry fasting

**Fig. 3:**
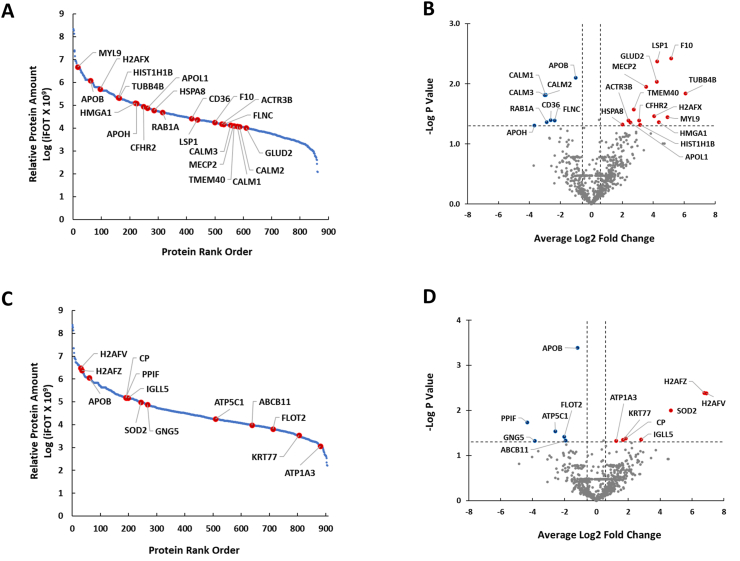
**Differentially Expressed Peripheral Blood Mononuclear Cell (PBMC) Gene Protein Products (GP) at the End of 4-Week Dawn-to-Dusk Dry Fasting and One Week after 4-Week Dawn-to-Dusk Dry Fasting**. **A.** The proteome coverage and its dynamic order from 14 samples taken at the end of 4-week dawn-to-dusk dry fasting. There were 862 cumulatively identified GPs recovered from before and at the end of 4-week dawn-to-dusk dry fasting samples with over six orders of magnitude of dynamic range. **B.** Volcano plot shows differentially expressed GPs that had greater than or equal to 1.5-fold significant change (blue and red represent downregulated and upregulated GPs, respectively) at the end of 4-week dawn-to-dusk dry fasting compared with the GP levels before 4-week dawn-to-dusk dry fasting. **C.** The proteome coverage and its dynamic order from 14 samples collected one week after 4-week dawn-to-dusk dry fasting. There were cumulatively 906 GPs recovered before and one week after 4-week dawn-to-dusk dry fasting with over six orders of magnitude of dynamic range. **D**. Volcano plot shows differentially expressed GPs that had greater than or equal to 1.5-fold significant change (blue and red represent downregulated and upregulated genes, respectively) one week after 4-week dawn-to-dusk dry fasting compared with the levels before 4-week dawn-to-dusk dry fasting. (For interpretation of the references to color in this figure legend, the reader is referred to the Web version of this article.)

The proteome coverage and its dynamic order from 14 samples taken at the end of 4-week dawn-to-dusk dry fasting are shown in Fig. 3A. There were 862 cumulatively identified GPs recovered from before and at the end of 4-week dawn-to-dusk dry fasting samples with over six orders of magnitude of dynamic range. Fig. 3A and B and Table 1 show the GPs that showed greater than or equal to 1.5-fold average paired change and a P-value of <0.05 at the end of 4-week dawn-to-dusk dry fasting compared with the levels before 4-week dawn-to-dusk dry fasting. There was an average 36 fold increase in coagulation factor X (F10) (log2 fold = 5.15, P = 0.004), 19 fold increase in lymphocyte specific protein 1 (LSP1) (log2 fold = 4.25, P = 0.004), 19 fold increase in glutamate dehydrogenase 2 (GLUD2) (log2 fold = 4.24, P = 0.009), 12 fold increase in methyl-CpG binding protein 2 (MECP2) (log2 fold = 3.54, P = 0.011), 68 fold increase in tubulin beta 4B class IVb (TUBB4B) (log2 fold = 6.08, P = 0.014), 7 fold increase in transmembrane protein 40 (TMEM40) (log2 fold = 2.74, P = 0.027), 17 fold increase in H2A.X variant histone (H2AFX) (log2 fold = 4.06, P = 0.035), 5 fold increase in actin related protein 3B (ACTR3B) (log2 fold = 2.39, P = 0.041), 8 fold increase in complement factor H related 2 (CFHR2) (log2 fold = 3.08, P = 0.041), 20 fold increase in high mobility group AT-hook 1 (HMGA1) (log2 fold = 4.36, P = 0.044), 6 fold increase in apolipoprotein L1 (APOL1) (log2 fold = 2.52, P = 0.044), 4 fold increase in heat shock protein family A (Hsp70) member 8 (HSPA8) (log2 fold = 2.01, P = 0.047), 9 fold increase in H1.5 linker histone, cluster member (HIST1H1B) (log2 fold = 3.16, P = 0.048) and 30 fold increase in myosin light chain 9 (MYL9) (log2 fold = 4.92, P = 0.036) GP levels at the end of 4-week dawn-to-dusk dry fasting compared with the levels before 4-week dawn-to-dusk dry fasting. There was a significant decrease in calmodulin 2 (CALM2) (log2 fold = −2.94, P = 0.015), calmodulin 1 (CALM1) (log2 fold = −2.96, P = 0.015), calmodulin 3 (CALM3) (log2 fold = −3.01, P = 0.015), CD36 molecule (CD36) (log2 fold = −2.63, P = 0.040), filamin C (FLNC) (log2 fold = −2.37, P = 0.041), RAB1A, member RAS oncogene family (RAB1A) (log2 fold = −2.88, P = 0.044), apolipoprotein H (APO) (log2 fold = −3.68, P = 0.0496) and apolipoprotein B (APOB) (log2 fold = −1.01, P = 0.008) GP levels at the end of 4-week dawn-to-dusk dry fasting compared with the levels before 4-week dawn-to-dusk dry fasting. Supplementary Table S1 shows the fold change in all GP levels at the end of 4-week dawn-to-dusk dry fasting compared with the GP levels before 4-week dawn-to-dusk dry fasting.

**Table 1:** Differentially expressed peripheral blood mononuclear cell (PBMC) gene protein products (GP) at the end of 4-week dawn-to-dusk dry fasting and one week after 4-week dawn-to-dusk dry fasting.

Gene Symbol	Gene ID	Gene Name	GP Levels at the End of 4-Week Dawn-to-Dusk Dry Fasting Compared with the GP Levels Before 4-Week Dawn-to-Dusk Dry Fasting		GP Levels One Week after 4-Week Dawn-to-Dusk Dry Fasting Compared with the GP Levels at the End of 4-Week Dawn-to-Dusk Dry Fasting		GP Levels One Week after 4-Week Dawn-to-Dusk Dry Fasting Compared with the GP Levels Before 4-Week Dawn-to-Dusk Dry Fasting	
			Average Paired Log2 Fold Change^a^	Paired P Value	Average Paired Log2 Fold Change^a^	Paired P Value	Average Paired Log2 Fold Change^a^	Paired P Value
**A1. GP Levels Upregulated at the End of 4-Week Dawn-to-Dusk Dry Fasting Compared with the GP Levels Before 4-Week Dawn-to-Dusk Dry Fasting**								
F10	2159	coagulation factor X	5.15	0.004	−2.96	0.026	2.19	0.207
LSP1	4046	lymphocyte specific protein	4.25	0.004	−2.34	0.062	1.92	0.284
GLUD2	2747	glutamate dehydrogenase 2	4.24	0.009	−2.18	0.038	2.06	0.175
MECP2	4204	methyl-CpG binding protein 2	3.54	0.011	−0.60	0.426	2.94	0.085
TUBB4B	10383	tubulin beta 4B class IVb	6.08	0.014	−2.35	0.255	3.73	0.076
TMEM40	55287	transmembrane protein 40	2.74	0.027	−1.04	0.541	1.70	0.295
H2AFX	3014	H2A.X variant histone	4.06	0.035	−1.36	0.434	2.71	0.259
ACTR3B	57180	actin related protein 3B	2.39	0.041	−1.10	0.393	1.29	0.167
CFHR2	3080	complement factor H related 2	3.08	0.041	−3.08	0.041	ND	ND
HMGA1	3159	high mobility group AT-hook 1	4.36	0.044	−1.94	0.252	2.42	0.270
APOL1	8542	apolipoprotein L1	2.52	0.044	−0.86	0.326	1.66	0.303
HSPA8	3312	heat shock protein family A (Hsp70) member 8	2.01	0.047	−0.61	0.419	1.41	0.322
HIST1H1B	3009	H1.5 linker histone, cluster member	3.16	0.048	−0.29	0.819	2.87	0.140
MYL9	10398	myosin light chain 9	4.92	0.036	−3.54	0.081	1.38	0.628
**A2. GP Levels Downregulated at the End of 4-Week Dawn-to-Dusk Dry Fasting Compared with the GP Levels Before 4-Week Dawn-to-Dusk Dry Fasting**								
CALM2	805	calmodulin 2	−2.94	0.015	1.59	0.160	−1.35	0.392
CALM1	801	calmodulin 1	−2.96	0.015	1.60	0.160	−1.36	0.393
CALM3	808	calmodulin 3	−3.01	0.015	1.63	0.161	−1.38	0.394
CD36	948	CD36 molecule	−2.63	0.040	1.85	0.083	−0.78	0.511
FLNC	2318	filamin C	−2.37	0.041	1.62	0.094	−0.75	0.611
RAB1A	5861	RAB1A, member RAS oncogene family	−2.88	0.044	2.21	0.120	−0.67	0.683
APOH	350	apolipoprotein H	−3.68	0.0496	3.06	0.071	−0.62	0.760
**B1. GP Levels Upregulated One Week after 4-Week Dawn-to-Dusk Dry Fasting Compared with the GP Levels Before 4-Week Dawn-to-Dusk Dry Fasting**								
H2AFZ	3015	H2A.Z variant histone 1	4.63	0.098	2.15	0.174	6.77	0.004
H2AFV	94239	H2A.Z variant histone 2	4.72	0.098	2.19	0.173	6.91	0.004
SOD2	6648	superoxide dismutase 2	0.51	0.690	4.17	0.013	4.68	0.010
CP	1356	ceruloplasmin	1.62	0.066	0.22	0.518	1.84	0.042
IGLL5	100423062	immunoglobulin lambda like polypeptide 5	2.25	0.221	0.56	0.649	2.81	0.044
KRT77	374454	keratin 77	ND	ND	1.67	0.045	1.67	0.045
ATP1A3	478	ATPase Na+/K+ transporting subunit alpha 3	0.95	0.093	0.30	0.686	1.25	0.048
**B2. GP Levels Downregulated One Week after 4-Week Dawn-to-Dusk Dry Fasting Compared with the GP Levels Before 4-Week Dawn-to-Dusk Dry Fasting**								
PPIF	10105	peptidylprolyl isomerase F	−2.59	0.233	−1.74	0.396	−4.33	0.019
ATP5C1	509	ATP synthase F1 subunit gamma	−0.04	0.982	−2.53	0.095	−2.57	0.029
FLOT2	2319	flotillin 2	−0.85	0.454	−1.16	0.193	−2.01	0.039
ABCB11	8647	ATP binding cassette subfamily B member 11	−1.18	0.312	−0.75	0.168	−1.93	0.047
GNG5	2787	G protein subunit gamma 5	−1.05	0.505	−2.80	0.119	−3.85	0.048
**C. GP Levels Downregulated Both at the End of 4-Week Dawn-to-Dusk Dry Fasting and One Week after 4-Week Dawn-to-Dusk Dry Fasting Compared with the GP Levels Before 4-Week Dawn-to-Dusk Dry Fasting**								
APOB	338	apolipoprotein B	−1.01	0.008	−0.16	0.559	−1.18	0.0004

ND=Not detected.

a A positive average paired log2 fold change indicates an increase, and a negative average paired log2 fold change indicates a decrease in the levels.

### 3.2 Changes in the gene protein products (GP) one week after 4-week dawn-to-dusk dry fasting compared with GP levels before 4-week dawn-to-dusk dry fasting

The proteome coverage and its dynamic order from 14 samples collected one week after 4-week dawn-to-dusk dry fasting are shown in Fig. 3C. There were cumulatively 906 GPs recovered before 4-week dawn-to-dusk dry fasting and one week after 4-week dawn-to-dusk dry fasting with over six orders of magnitude of dynamic range. Fig. 3C and D and Table 1 show the GPs that showed an equal to or greater than 1.5-fold average paired change and a P-value of <0.05 one week after 4-week dawn-to-dusk dry fasting compared with GP levels before 4-week dawn-to-dusk dry fasting. There was an average 109 fold increase in H2A.Z variant histone 1 (H2AFZ) (log2 fold = 6.77, P = 0.004), 120 fold increase in H2A.Z variant histone 2 (H2AFV) (log2 fold = 6.91, P = 0.004), 26 fold increase in superoxide dismutase 2 (SOD2) (log2 fold = 4.68, P = 0.010), 4 fold increase in ceruloplasmin (CP) (log2 fold = 1.84, P = 0.042), 7 fold increase in immunoglobulin lambda like polypeptide 5 (IGLL5) (log2 fold = 2.81, P = 0.044), 3 fold increase in keratin 77 (KRT77) (log2 fold = 1.67, P = 0.045), and 2 fold increase in ATPase Na+/K+ transporting subunit alpha 3 (ATP1A3) (log2 fold = 1.25, P = 0.048) GP levels one week after 4-week dawn-to-dusk dry fasting compared with the levels before 4-week dawn-to-dusk dry fasting. There was a significant decrease in peptidylprolyl isomerase F (PPIF) (log2 fold = −4.33, P = 0.019), ATP synthase F1 subunit gamma (ATP5C1) (log2 fold = −2.57, P = 0.029), flotillin 2 (FLOT2) (log2 fold = −2.01, P = 0.039), ATP binding cassette subfamily B member 11 (ABCB11) (log2 fold = −1.93, P = 0.047), G protein subunit gamma 5 (GNG5) (log2 fold = −3.85, P = 0.048, and apolipoprotein B (APOB) (log2 fold = −1.18, P = 0.0004) in GP levels one week after 4-week dawn-to-dusk dry fasting compared with the levels before 4-week dawn-to-dusk dry fasting. Supplementary Table S2 shows the fold change in all GP levels one week after 4-week dawn-to-dusk dry fasting compared with the GP levels before 4-week dawn-to-dusk dry fasting.

### 3.3 Changes in the gene protein products (GP) one week after 4-week dawn-to-dusk dry fasting compared with GP levels at the end of 4-week dawn-to-dusk dry fasting

There was a significant average paired fold change in the levels of several GPs one week after 4-week dawn-to-dusk dry fasting compared with the levels at the end of 4-week dawn-to-dusk dry fasting (Supplementary Table S3). Supplementary Table 3 also shows the fold change in all GP levels one week after 4-week dawn-to-dusk dry fasting compared with the GP levels at the end of 4-week dawn-to-dusk dry fasting. Table 1 shows some of these GPs that showed differential expression.

### 3.4 Pathways and biological processes associated with differentially expressed genes comparing gene protein products (GP) levels at the end of 4-week dawn-to-dusk dry fasting with GP levels before 4-week dawn-to-dusk dry fasting

**Fig. 4:**
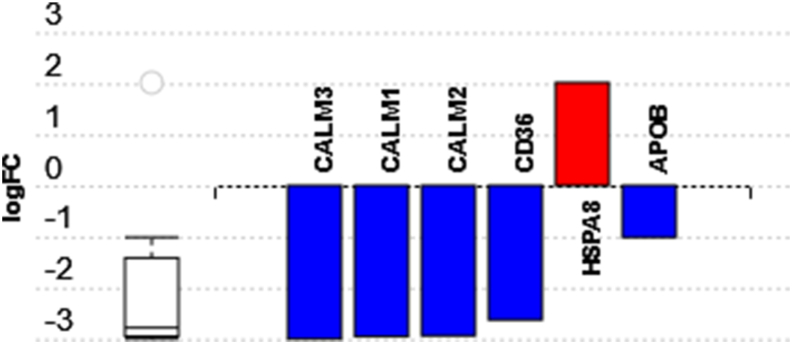
**The anti-atherosclerotic proteome induced by dawn-to-dusk dry fasting.** This bar plot displays the differentially expressed genes associated with lipid and atherosclerosis pathway comparing GP levels at the end of 4-week dawn-to-dusk fasting with the pre-fasting levels (blue and red represent downregulated and upregulated genes, respectively). The number of differentially expressed genes/number of all genes was 6/32, with a P = 6.014e-4 (FC = fold change). Apolipoprotein B (APOB) GP levels were downregulated along with CALM1, CALM2, CALM3 and CD36 GP levels and had the most statistical significance of the observed difference at the end of 4-week dawn-to-dusk dry fasting (P = 0.008). Fig. 4 was obtained using Advaita Bio's iPathwayGuide [27], [28], [29], [30] (For interpretation of the references to color in this figure legend, the reader is referred to the Web version of this article.)

Twenty-two differentially expressed genes were identified (Table 1). The top three pathways that were associated with the highest number of differentially expressed genes were lipid and atherosclerosis pathway (number of differentially expressed genes/number of all genes = 6/32, P = 6.014e-4) (Fig. 4), pathways of neurodegeneration-multiple diseases (number of differentially expressed genes/number of all genes = 5/76, P = 0.004), and C-type lectin receptor signaling pathway (number of differentially expressed genes/number of all genes = 4/8, P = 1.064e-5). The C-type lectin receptor signaling pathway was the most impacted pathway. The top Gene Ontology (GO) term biological process associated with the highest number of differentially expressed genes was response to stimulus (number of differentially expressed genes/number of all genes = 17/478, P = 0.027).

### 3.5 Pathways and biological processes associated with differentially expressed genes comparing gene protein products (GP) levels one week after 4-week dawn-to-dusk dry fasting with GP levels at the end of 4-week dawn-to-dusk dry fasting

Thirteen differentially expressed genes were identified (Table 1). The top four pathways that were associated with the highest number of differentially expressed genes were chemical carcinogenesis-reactive oxygen species (number of differentially expressed genes/number of all genes = 3/41, P = 0.019), Huntington disease (number of differentially expressed genes/number of all genes = 3/57, P = 0.025), bile secretion (number of differentially expressed genes/number of all genes = 2/6, P = 0.004) and cholesterol metabolism (number of differentially expressed genes/number of all genes = 2/14, P = 0.023). Bile secretion was also the most impacted pathway (P = 0.004), followed by chemical carcinogenesis - reactive oxygen species (P = 0.019) and cholesterol metabolism (P = 0.023). The top GO term biological process associated with the highest number of differentially expressed genes was cellular response to stimulus (number of differentially expressed genes/number of all genes = 9/353, P = 0.038).

### 3.6 Correlations between PBMC GP levels and metabolic syndrome components, lipid panel, hepatic panel, adiposity, oxidative stress, and inflammation biomarkers

We previously reported the effect of 4-week dawn-to-dusk dry fasting on the same subjects’ metabolic syndrome components (e.g., weight, waist circumference, blood pressure, glucose), insulin, lipid panel, hepatic panel, and adiposity, oxidative stress, and inflammation biomarker levels [24]. Subjects had a significant reduction in weight, body mass index, waist circumference, systolic, diastolic, and mean arterial blood pressures at the end of 4-week dawn-to-dusk dry fasting and a significant reduction in weight, body mass index, waist circumference, and homeostatic model assessment for insulin resistance (HOMA-IR) one week after 4-week dawn-to-dusk dry compared with the levels before fasting as previously reported [24]. In the present study, we found several significant correlations between fold changes in the metabolic syndrome components, lipid panel, hepatic panel, adiposity, oxidative stress, and inflammation biomarker levels and fold changes in PBMC GP levels at the end of 4-week dawn-to-dusk dry fasting and one week after 4-week dawn-to-dusk dry fasting compared with GP levels before 4-week dawn-to-dusk dry fasting (Supplementary Tables S4 and S5).

## 4 Discussion

This study showed that subjects with metabolic syndrome who subscribed to the dawn-to-dusk dry fasting had anti-atherosclerotic, anti-inflammatory, and anti-tumorigenic PMBC proteome. The proteomic changes in PBMC induced by 4-week dawn-to-dusk dry fasting were similar to the proteomic changes in serum induced by 4-week dawn-to-dusk dry fasting observed in the same subjects [24]. Additionally, dry fasting from dawn to dusk for four weeks was feasible and safe in the study subjects.

### 4.1 Four-week dawn-to-dusk dry fasting induced differential expression of genes on lipid and atherosclerosis pathway

Six differentially expressed genes on the lipid and atherosclerosis pathway (number of differentially expressed genes/number of all genes = 6/32, P = 6.014e-4) were identified when GPs at the end of 4-week dawn-to-dusk dry fasting were compared with the GP levels before fasting (Fig. 4). One of these genes, APOB, encodes for apolipoprotein B which is the primary apolipoprotein in the low-density lipoprotein cholesterol and is associated with atherosclerosis [33,34]. Atherosclerosis, inflammation of the arterial wall intima, is the leading cause of atherosclerotic cardiovascular disease [35,36]. Apolipoprotein B was shown to be a more accurate risk predictor for developing atherosclerotic heart disease than low-density lipoprotein cholesterol [37]. Our results showed that APOB GP level was significantly downregulated at the end of 4-week dawn-to-dusk dry fasting compared with the GP level before 4-week dawn-to-dusk dry fasting, and its downregulation persisted even one week after 4-week dawn-to-dusk dry fasting.

CD36 was also differentially expressed and downregulated on the lipid and atherosclerosis pathway (Fig. 4). CD36, a glycoprotein located on the surface of platelets, macrophages, monocytes, adipocytes, and endothelial cells, is considered a significant player in the development of atherosclerosis [38]. The binding of CD36 with the oxidized low-density lipoprotein results in the trapping of the macrophages in the arterial intima and induces inflammatory processes leading to luminal stenosis [38].

Similar to APOB and CD36, the CALM1, CALM2, and CALM3 encoding calmodulin 1, 2, and 3, respectively [[39], [40], [41]] were differentially expressed and downregulated on the lipid and atherosclerosis pathway (Fig. 4). Smooth muscle cells are known to play a significant role in the development of atherosclerosis [[42], [43], [44]]. An increased calmodulin expression was observed in atheroma-susceptible small smooth muscle cells derived from human carotid endarterectomy specimens, and a calmodulin inhibitor was able to reduce small smooth muscle cell proliferation, indicating the key role of calmodulin in the formation of atherosclerotic plaque [44].

A mean 4-fold increase in the GP of HSPA8, also known as HSP70 [45], was found at the end of 4-week dawn-to-dusk dry fasting compared with the GP levels before 4-week dawn-to-dusk dry fasting (Table 1). In contrast to several other heat shock proteins, the upregulation of HSPA8 was shown to have a protective role against atherosclerosis [46,47]. A study conducted on 421 patients showed that subjects without coronary artery disease had significantly elevated HSP70 levels compared with those diagnosed with coronary artery disease [47]. The same study also showed that subjects with elevated HSP70 levels had a nearly 50% reduced risk of developing coronary artery disease than subjects with lower HSP70 levels after adjusting for traditional coronary artery disease risk factors [47].

Altogether, our results suggest that 4-week dawn-to-dusk dry fasting should be considered in preventing and treating atherosclerotic cardiovascular disease.

### 4.2 Four-week dawn-to-dusk dry fasting induced differential expression of genes on C-type lectin receptor signaling pathway

C-type lectin receptors expressed by antigen-presenting cells (i.e., dendritic cells, macrophages, and Langerhans cells) are pattern recognition receptors that play a significant role in the recognition and presentation of antigens, induction of T helper cell differentiation and innate and adaptive immune responses against pathogens [48]. Four differentially expressed genes associated with the C-type lectin receptor signaling pathway (number of differentially expressed genes/number of all genes = 4/8, P = 1.064e-5) were identified. One of the differentially expressed genes on the C-type lectin receptor signaling pathway was LSP1. An average 19-fold increase in LSP1 GP levels was found at the end of 4-week dawn-to-dusk dry fasting compared with the GP levels before 4-week dawn-to-dusk dry fasting (Table 1). LSP1 gene that encodes F-actin binding protein in the cytoskeleton of leukocytes (e.g., neutrophils, lymphoctes, macrophages, monocytes, dendritic cells) and endothelium, regulates leukocyte migration and adhesion, specifically during inflammation [[49], [50], [51]]. High levels of LSP1 downregulate leukocyte migration to the inflammation sites by inhibiting adhesion and superoxide generation mediated by Mac-1, whereas low levels of LSP1 upregulate leukocyte migration [51]. Multiple studies of dawn-to-dusk dry fasting showed an anti-inflammatory effect of dawn-to-dusk dry fasting [52,53]. Our findings showed upregulation of the LSP1 gene at the end of 4-week dawn-to-dusk dry fasting, which could be one of the mechanisms behind the anti-inflammatory effect of dawn-to-dusk dry fasting.

### 4.3 Four-week dawn-to-dusk dry fasting induced a proteome associated with protection against autoimmunity

Similar to the inflammatory effect of LSP1 deficiency, MECP2 deficiency also was shown to increase inflammation. MECP2 deficiency results in the expression of inflammatory cytokines, including tumor necrosis factor-α, interleukin-6, and interleukin-3, by activating the NF-κB signaling pathway in PBMC [54]. In fact, MECP2 plays a critical role in the suppression of inflammation and protection against autoimmunity by maintaining a stable Foxp3 expression in T regulatory cells [55]. We found an average 12-fold increase in MECP2 levels at the end of 4-week dawn-to-dusk dry fasting compared with the levels before fasting.

### 4.4 Four-week dawn-to-dusk dry fasting induced a proteome associated with protection against several cancers

Our previous studies showed that 4-week dawn-to-dusk dry fasting induced an anti-tumorigenic proteome in the serum by up- or down-regulating several proteins playing a key role in carcinogenesis [23,24]. Likewise, 4-week dawn-to-dusk dry fasting induced an anti-tumorigenic proteome in PBMC. We found an average 19-fold increase in LSP1 GP levels at the end of 4-week dawn-to-dusk dry fasting compared with the GP levels before 4-week dawn-to-dusk dry fasting (Table 1). Among subjects with hepatocellular carcinoma, LSP1 was downregulated, and those with higher LSP1 expression had a higher 5-year overall and disease-free survival compared with subjects with lower LSP1 expression even after controlling for tumor size, histological grading, and TNM tumor stage [56]. This knowledge is important because patients with metabolic syndrome are at risk for developing hepatocellular carcinoma in the background of fatty liver disease-induced cirrhosis [8].

TUBB4 downregulation and upregulation of TUBB3 results in epithelial-mesenchymal transition of tumor cells and tumor metastasis in colon cancer [57]. TUBB4 downregulation was also shown in taxane-resistant breast cancer compared with taxane-sensitive breast cancer [58]. We found an average 68-fold increase in the TUBB4 GP levels at the end of 4-week dawn-to-dusk dry fasting compared with the GP levels before fasting (Table 1).

Similar to the upregulation of LSP1 and TUBB4 GPs, we observed differential expression of ACTR3B at the end of 4-week dawn-to-dusk dry fasting compared with baseline. ACTR3B, also known as ARP11, encodes actin-related proteins that play a key role in the regulation of cell motility [59]. Upregulation of ACTRB3 was shown to suppress the growth of PC-14 lung adenocarcinoma cells [60].

The observed downregulation of CD36, CALM1, 2, and 3 GP levels at the end of 4-week dawn-to-dusk dry fasting and FLOT2 one week after 4-week dawn-to-dusk dry fasting compared with GP levels before fasting contributes to the anti-tumorigenic effect of dawn-to-dusk dry fasting along with changes in the expression of other genes. Calmodulin which is encoded by CALM1, CALM2, and CALM3 genes [[39], [40], [41]], plays a significant role in non-tumor cell migration as well as tumor migration and metastasis [61]. Several anti-cancer agents with antagonistic action for calmodulin were reported to prevent tumor progression and metastasis [61]. Overexpression of CD36 was reported in tumor metastasis of various cancers (e.g., breast cancer, ovarian cancer, pancreatic ductal adenocarcinoma) [[62], [63], [64], [65]]. Likewise, overexpression of FLOT2 was shown to result in epithelial-mesenchymal transition of tumor cells and, thereby, metastasis in hepatocellular carcinoma [66] and overexpression of PPIF was reported to be a poor prognostic sign in endometrial cancer [67].

## 5 Strengths and limitations

The main strength of the study is that this is the first study that examined PBMC proteomic changes in the same subjects before, during and after the dawn-to-dusk dry fasting. Although this study was limited to 14 subjects with metabolic syndrome and lacked a non-fasting control group, a robust proteomics method was used to demonstrate the anti-atherosclerotic, anti-inflammatory, and anti-tumorigenic effects of 4-week dawn-to-dusk dry fasting in PBMC. These findings contribute to previous serum proteome changes induced by 4-week dawn-to-dusk dry fasting in the same subjects [24] and confirm the proteomic changes induced by dawn-to-dusk dry fasting at the cellular level (PMBC). PBMC proteomics provided us with a mechanistic insight into protein expression changes without a tissue biopsy. The changes observed in serum and PBMC at the end of 4-week dawn-to-dusk dry fasting and one week after 4-week dawn-to-dusk dry fasting should be considered a strong indication of how the proteome changed at the tissue level.

## 6 Conclusions

Based on previous reports [23,24,68] and the findings of this study, dawn-to-dusk dry fasting appears to be a potential intervention in the prevention and treatment of several chronic metabolic diseases and metabolic syndrome-induced cancers. Dawn-to-dusk dry fasting can also be an ultimate intervention to reset the inner circadian clock and align both central and peripheral clocks with the local dawn and dusk time. Randomized controlled clinical trials with integrative multi-omics analysis (e.g., circadian transcriptomics, metabolomics, proteomics) are needed to investigate the effect of dawn-to-dusk dry fasting in subjects with chronic metabolic diseases and metabolic syndrome-induced cancers before any recommendations can be made.

## Funding

This project was supported by the 2019 Roderick D. MacDonald Research Award/10.13039/100007492Baylor St. Luke's Medical Center (Award Number 19RDM001) (to Ayse L. Mindikoglu, MD, MPH) and 2020 Roderick D. MacDonald Research Award/10.13039/100007492Baylor St. Luke's Medical Center (Award Number 20RDM006) (to Ayse L. Mindikoglu, MD, MPH). This project was also supported in part by NIH Public Health Service grant P30DK056338, which funds the Texas Medical Center Digestive Diseases Center, and its contents are solely the responsibility of the authors and do not necessarily represent the official views of the 10.13039/100000062National Institute of Diabetes and Digestive and Kidney Diseases or the NIH. Additional support was provided by a gift from the D.R. and G.P. Laws Fund which supports the Opekun G.I. Research Laboratory.

## Conflicts of interest

None of the authors has a conflict of interest.

## Credit authorship contribution statement

**Ayse L. Mindikoglu:** formulated the study hypothesis and study concept, designed the study, drafted and wrote the manuscript, contributed with conducting the study, analyzing data, and critically reviewing and finalizing the manuscript. **Jihwan Park:** contributed with performing PBMC proteomic analysis and critically reviewing the manuscript. **Antone R. Opekun:** contributed with conducting the study, and critically reviewing the manuscript. **Mustafa M. Abdulsada:** contributed with conducting the study, and critically reviewing the manuscript. **Zoe R. Wilhelm:** contributed with conducting the study and critically reviewing the manuscript. **Prasun K. Jalal:** contributed with conducting the study, and critically reviewing the manuscript. **Sridevi Devaraj:** contributed with performing the analysis of components of metabolic syndrome, lipid and hepatic panels, and adiposity, oxidative stress and inflammation biomarkers and critically reviewing the manuscript. **Sung Yun Jung:** contributed with performing PBMC proteomic analysis, analyzing data, writing the proteomic method section, and critically reviewing the manuscript.
