# Supplementary material for: Dawn-to-dusk dry fasting induces anti-atherosclerotic, anti-inflammatory, and anti-tumorigenic proteome in peripheral blood mononuclear cells in subjects with metabolic syndrome
Source: Metabol Open. 2022 Nov 1;16:100214. doi: 10.1016/j.metop.2022.100214 (PMC9731888; doi:10.1016/j.metop.2022.100214)
Supplement: Supplementary Table S2 [file mmc2.docx]

| **Supplementary Table S2. Gene Protein Product (GP) Levels that Are Up- or Downregulated One Week after 4-Week Dawn-to-Dusk Dry Fasting (V3) Compared with the GP Levels Before 4-Week Dawn-to-Dusk Dry Fasting (V1)** | | | |
| --- | --- | --- | --- |
| **Gene Symbol** | **Gene ID** | **Average Paired Log2 Fold Change (V3/V1)** | **Paired P Value** |
| H2AFZ | 3015 | 6.77 | 0.0041 |
| H2AFV | 94239 | 6.91 | 0.0042 |
| SOD2 | 6648 | 4.68 | 0.0100 |
| CP | 1356 | 1.84 | 0.0416 |
| IGLL5 | 100423062 | 2.81 | 0.0438 |
| KRT77 | 374454 | 1.67 | 0.0447 |
| ATP1A3 | 478 | 1.25 | 0.0475 |
| APOB | 338 | -1.18 | 0.0004 |
| PPIF | 10105 | -4.33 | 0.0186 |
| ATP5C1 | 509 | -2.57 | 0.0289 |
| FLOT2 | 2319 | -2.01 | 0.0386 |
| ABCB11 | 8647 | -1.93 | 0.0468 |
| GNG5 | 2787 | -3.85 | 0.0475 |
| ABRACL | 58527 | 2.21 | 0.0524 |
| SERPINF2 | 5345 | 2.24 | 0.0683 |
| TUFM | 7284 | 1.83 | 0.0740 |
| HNRNPH2 | 3188 | 1.27 | 0.0831 |
| MECP2 | 4204 | 2.94 | 0.0847 |
| VAMP8 | 8673 | 2.04 | 0.0870 |
| FHOD1 | 29109 | 0.81 | 0.0883 |
| PGLYRP2 | 114770 | 2.48 | 0.0963 |
| MYH4 | 4622 | -1.89 | 0.0576 |
| PDHA1 | 5160 | -1.61 | 0.0785 |
| SDR42E1 | 93517 | -3.64 | 0.0803 |
| HVCN1 | 84329 | -1.48 | 0.0834 |
| ATP2A1 | 487 | -1.26 | 0.0834 |
| LIMS1 | 3987 | -1.72 | 0.0857 |
| PARVA | 55742 | -1.45 | 0.0860 |
| RPS19 | 6223 | -2.07 | 0.0868 |
| ITGB2 | 3689 | -1.35 | 0.0906 |
| FAM162A | 26355 | -2.07 | 0.0919 |
| PPBP | 5473 | -0.53 | 0.0999 |
| TF | 7018 | 0.45 | 0.0468 |
| HP | 3240 | 0.60 | 0.0620 |
| ALB | 213 | 0.35 | 0.0728 |
| TUBB4B | 10383 | 3.73 | 0.0762 |
| FCER1G | 2207 | 2.76 | 0.0803 |
| YWHAH | 7533 | 2.62 | 0.0919 |
| FGA | 2243 | -0.29 | 0.0941 |
| AGT | 183 | 2.13 | 0.0995 |
| RAB10 | 10890 | -2.93 | 0.1004 |
| CKM | 1158 | -2.04 | 0.1012 |
| CAP1 | 10487 | 2.47 | 0.1052 |
| PLCG1 | 5335 | -4.04 | 0.1079 |
| NDUFA5 | 4698 | -2.83 | 0.1088 |
| GSTK1 | 373156 | -1.73 | 0.1088 |
| CPN1 | 1369 | 2.17 | 0.1096 |
| MYH6 | 4624 | -1.64 | 0.1126 |
| MYH7 | 4625 | -1.65 | 0.1126 |
| H2AFY | 9555 | 3.42 | 0.1135 |
| PON1 | 5444 | 2.20 | 0.1189 |
| ACTG2 | 72 | 4.53 | 0.1201 |
| ACTA2 | 59 | 4.48 | 0.1203 |
| CLEC3B | 7123 | -3.06 | 0.1214 |
| C1R | 715 | -2.46 | 0.1245 |
| GP9 | 2815 | 2.60 | 0.1261 |
| C4B_2 | 100293534 | 0.40 | 0.1277 |
| C4B | 721 | 0.40 | 0.1277 |
| HLA-B | 3106 | 1.90 | 0.1279 |
| CHCHD2 | 51142 | 2.67 | 0.1316 |
| AMBP | 259 | 1.85 | 0.1323 |
| TMSB4X | 7114 | 2.66 | 0.1348 |
| ITIH1 | 3697 | 0.33 | 0.1355 |
| ACTB | 60 | -0.62 | 0.1363 |
| HIST1H1B | 3009 | 2.87 | 0.1402 |
| YWHAB | 7529 | 2.57 | 0.1419 |
| RTN4 | 57142 | 1.60 | 0.1482 |
| PTPRC | 5788 | 1.96 | 0.1520 |
| ZMPSTE24 | 10269 | -1.68 | 0.1526 |
| ACTBL2 | 345651 | -4.84 | 0.1528 |
| HIST1H1C | 3006 | 1.66 | 0.1529 |
| DLST | 1743 | 1.44 | 0.1555 |
| ACTG1 | 71 | 2.77 | 0.1572 |
| RAB6B | 51560 | -2.00 | 0.1580 |
| EHD1 | 10938 | 0.66 | 0.1596 |
| RTN3 | 10313 | 0.89 | 0.1613 |
| ATP1A1 | 476 | 1.09 | 0.1642 |
| CISD2 | 493856 | -1.19 | 0.1648 |
| PLP2 | 5355 | 1.62 | 0.1648 |
| TMX3 | 54495 | -0.96 | 0.1648 |
| GP6 | 51206 | 1.00 | 0.1648 |
| RPL10A | 4736 | 0.91 | 0.1648 |
| ACTRT1 | 139741 | 1.67 | 0.1648 |
| COX5A | 9377 | 1.50 | 0.1649 |
| S100A4 | 6275 | 2.28 | 0.1650 |
| PHKB | 5257 | -1.32 | 0.1650 |
| CDC42 | 998 | 1.41 | 0.1651 |
| GPX3 | 2878 | 1.08 | 0.1651 |
| PTPRCAP | 5790 | 1.10 | 0.1651 |
| EIF2S1 | 1965 | -0.83 | 0.1652 |
| TAS2R42 | 353164 | -1.17 | 0.1652 |
| DLD | 1738 | 1.11 | 0.1652 |
| MGST2 | 4258 | 1.29 | 0.1652 |
| SNCA | 6622 | 0.98 | 0.1654 |
| RPL29 | 6159 | -1.37 | 0.1655 |
| KIT | 3815 | 1.13 | 0.1655 |
| LTN1 | 26046 | 1.41 | 0.1656 |
| 44445 | 23157 | 0.94 | 0.1657 |
| DBNL | 28988 | 0.80 | 0.1658 |
| TWF2 | 11344 | -0.94 | 0.1659 |
| TECR | 9524 | 0.98 | 0.1661 |
| RPL9 | 6133 | -1.29 | 0.1665 |
| APCS | 325 | 1.10 | 0.1665 |
| HSPA5 | 3309 | 1.44 | 0.1671 |
| DES | 1674 | -1.32 | 0.1671 |
| ACTR3B | 57180 | 1.29 | 0.1675 |
| CYBA | 1535 | 1.41 | 0.1676 |
| CST3 | 1471 | -1.13 | 0.1683 |
| DIAPH1 | 1729 | -0.74 | 0.1684 |
| PDE5A | 8654 | 0.81 | 0.1684 |
| RPL4 | 6124 | -2.08 | 0.1684 |
| NDUFB10 | 4716 | -1.14 | 0.1692 |
| SORCS3 | 22986 | -1.22 | 0.1694 |
| NAPA | 8775 | 1.02 | 0.1696 |
| OGDHL | 55753 | -1.13 | 0.1697 |
| F12 | 2161 | 0.87 | 0.1698 |
| ACAA2 | 10449 | 1.07 | 0.1699 |
| PNP | 4860 | -1.49 | 0.1701 |
| ABHD16A | 7920 | 0.96 | 0.1711 |
| TUBB1 | 81027 | 1.34 | 0.1720 |
| CAPN1 | 823 | 0.91 | 0.1720 |
| PDIA3 | 2923 | 1.49 | 0.1726 |
| TMED2 | 10959 | 0.97 | 0.1734 |
| RAP1A | 5906 | -1.15 | 0.1740 |
| GLUD2 | 2747 | 2.06 | 0.1754 |
| FGG | 2266 | -0.19 | 0.1755 |
| FLNA | 2316 | 0.46 | 0.1764 |
| RPL26L1 | 51121 | 1.29 | 0.1774 |
| RPL26 | 6154 | 1.29 | 0.1774 |
| LMAN2 | 10960 | 0.12 | 0.1782 |
| MYL5 | 4636 | 1.28 | 0.1783 |
| SUN2 | 25777 | 1.05 | 0.1799 |
| ACTR1A | 10121 | -1.78 | 0.1817 |
| SQRDL | 58472 | -0.84 | 0.1819 |
| ORMDL3 | 94103 | 1.69 | 0.1839 |
| DECR1 | 1666 | -1.71 | 0.1849 |
| LBR | 3930 | -1.11 | 0.1854 |
| PARK7 | 11315 | -1.16 | 0.1868 |
| UBC | 7316 | 1.67 | 0.1874 |
| ITIH4 | 3700 | 1.40 | 0.1887 |
| ARPC3 | 10094 | -1.94 | 0.1899 |
| KIAA1522 | 57648 | -0.98 | 0.1904 |
| HSPE1-MOB4 | 100529241 | -1.31 | 0.1910 |
| RPL27A | 6157 | -1.11 | 0.1927 |
| PDLIM1 | 9124 | -2.52 | 0.1934 |
| CFI | 3426 | 1.63 | 0.1953 |
| TMX4 | 56255 | -0.93 | 0.1955 |
| TPM3 | 7170 | 1.70 | 0.2001 |
| JCHAIN | 3512 | 2.09 | 0.2020 |
| CFB | 629 | 1.29 | 0.2042 |
| UBB | 7314 | 1.90 | 0.2063 |
| ORM1 | 5004 | 1.66 | 0.2065 |
| NEXN | 91624 | 0.91 | 0.2067 |
| F10 | 2159 | 2.19 | 0.2070 |
| ECH1 | 1891 | -2.13 | 0.2102 |
| TUBA8 | 51807 | 2.08 | 0.2104 |
| PON3 | 5446 | -1.77 | 0.2115 |
| GP1BA | 2811 | 1.55 | 0.2123 |
| A2M | 2 | 1.13 | 0.2143 |
| UBA52 | 7311 | 2.04 | 0.2156 |
| APOC1 | 341 | 1.82 | 0.2157 |
| CLU | 1191 | -0.31 | 0.2168 |
| RPS27A | 6233 | 2.08 | 0.2175 |
| TUBA4A | 7277 | 1.34 | 0.2177 |
| LTBP1 | 4052 | 1.55 | 0.2184 |
| CFH | 3075 | 0.38 | 0.2211 |
| PPIA | 5478 | -1.95 | 0.2242 |
| SEC23A | 10484 | -1.84 | 0.2243 |
| M6PR | 4074 | 1.14 | 0.2244 |
| SFXN1 | 94081 | 1.51 | 0.2252 |
| TST | 7263 | -1.92 | 0.2262 |
| RBP4 | 5950 | 2.16 | 0.2316 |
| RPL23A | 6147 | -2.42 | 0.2348 |
| TUBB | 203068 | 1.51 | 0.2367 |
| PPA2 | 27068 | 1.52 | 0.2396 |
| ITIH2 | 3698 | 1.15 | 0.2407 |
| HSPA1L | 3305 | 0.06 | 0.2407 |
| CALD1 | 800 | 1.75 | 0.2407 |
| RAB7A | 7879 | -1.48 | 0.2408 |
| IQGAP2 | 10788 | 0.92 | 0.2413 |
| TPM1 | 7168 | 1.90 | 0.2422 |
| RDH11 | 51109 | 1.34 | 0.2478 |
| TTR | 7276 | 0.41 | 0.2490 |
| TUBA4B | 80086 | 2.21 | 0.2504 |
| SAA4 | 6291 | 2.32 | 0.2536 |
| H2AFX | 3014 | 2.71 | 0.2585 |
| HIST1H2AA | 221613 | 2.75 | 0.2587 |
| KRT1 | 3848 | -1.14 | 0.2609 |
| YWHAG | 7532 | 2.27 | 0.2617 |
| MGST3 | 4259 | 0.54 | 0.2624 |
| MAOB | 4129 | 1.33 | 0.2624 |
| ANXA2 | 302 | 1.79 | 0.2695 |
| HMGA1 | 3159 | 2.42 | 0.2699 |
| APOL3 | 80833 | 1.87 | 0.2701 |
| SERPINC1 | 462 | 0.96 | 0.2702 |
| CECR5 | 27440 | 0.76 | 0.2714 |
| CHMP4A | 29082 | -1.07 | 0.2729 |
| GP5 | 2814 | -1.54 | 0.2763 |
| HSP90B1 | 7184 | 1.02 | 0.2802 |
| HSPA1A | 3303 | 1.06 | 0.2805 |
| HSPA1B | 3304 | 1.06 | 0.2805 |
| DLAT | 1737 | 0.47 | 0.2819 |
| SERPIND1 | 3053 | 1.37 | 0.2834 |
| LMNB2 | 84823 | 0.70 | 0.2840 |
| LSP1 | 4046 | 1.92 | 0.2843 |
| SRPRB | 58477 | 1.08 | 0.2874 |
| S100A10 | 6281 | 1.73 | 0.2900 |
| COX2 | 4513 | 0.75 | 0.2905 |
| C5 | 727 | 1.31 | 0.2928 |
| HIST4H4 | 121504 | 1.17 | 0.2951 |
| HIST2H4B | 554313 | 1.17 | 0.2951 |
| HIST1H4I | 8294 | 1.17 | 0.2951 |
| HIST1H4A | 8359 | 1.17 | 0.2951 |
| HIST1H4D | 8360 | 1.17 | 0.2951 |
| HIST1H4F | 8361 | 1.17 | 0.2951 |
| HIST1H4K | 8362 | 1.17 | 0.2951 |
| HIST1H4J | 8363 | 1.17 | 0.2951 |
| HIST1H4C | 8364 | 1.17 | 0.2951 |
| HIST1H4H | 8365 | 1.17 | 0.2951 |
| HIST1H4B | 8366 | 1.17 | 0.2951 |
| HIST1H4E | 8367 | 1.17 | 0.2951 |
| HIST1H4L | 8368 | 1.17 | 0.2951 |
| HIST2H4A | 8370 | 1.17 | 0.2951 |
| TMEM40 | 55287 | 1.70 | 0.2955 |
| APOA4 | 337 | 1.19 | 0.2981 |
| TMX1 | 81542 | 1.51 | 0.2984 |
| SDPR | 8436 | 1.45 | 0.3004 |
| PF4 | 5196 | 1.38 | 0.3011 |
| PF4V1 | 5197 | 1.38 | 0.3011 |
| ERP29 | 10961 | 1.38 | 0.3015 |
| APOL1 | 8542 | 1.66 | 0.3031 |
| RPN1 | 6184 | -1.38 | 0.3049 |
| AHSG | 197 | 1.74 | 0.3071 |
| NDUFV2 | 4729 | 1.11 | 0.3072 |
| TXN | 7295 | 1.52 | 0.3089 |
| ARPC5 | 10092 | -1.83 | 0.3110 |
| HNRNPA2B1 | 3181 | 1.38 | 0.3117 |
| HIST1H1E | 3008 | -2.58 | 0.3124 |
| YWHAQ | 10971 | 1.20 | 0.3133 |
| PDHB | 5162 | 1.12 | 0.3139 |
| SLC2A3 | 6515 | 1.41 | 0.3142 |
| HPX | 3263 | 1.17 | 0.3175 |
| P4HB | 5034 | 1.45 | 0.3185 |
| ARL8A | 127829 | -0.47 | 0.3206 |
| C1S | 716 | 0.94 | 0.3213 |
| HSPA8 | 3312 | 1.41 | 0.3219 |
| RAC1 | 5879 | -0.61 | 0.3232 |
| RAC2 | 5880 | -0.63 | 0.3236 |
| RAC3 | 5881 | -0.63 | 0.3236 |
| SNAP23 | 8773 | 1.84 | 0.3240 |
| LOC102724334 | 102724334 | 1.19 | 0.3241 |
| H2BFS | 54145 | 1.19 | 0.3241 |
| HIST1H2BG | 8339 | 1.19 | 0.3241 |
| HIST1H2BN | 8341 | 1.19 | 0.3241 |
| HIST1H2BM | 8342 | 1.19 | 0.3241 |
| HIST1H2BF | 8343 | 1.19 | 0.3241 |
| HIST1H2BE | 8344 | 1.19 | 0.3241 |
| HIST1H2BH | 8345 | 1.19 | 0.3241 |
| HIST1H2BI | 8346 | 1.19 | 0.3241 |
| HIST1H2BC | 8347 | 1.19 | 0.3241 |
| HIST1H2BK | 85236 | 1.19 | 0.3241 |
| HIST1H2BD | 3017 | 1.19 | 0.3243 |
| HIST2H2BF | 440689 | 1.19 | 0.3243 |
| PLG | 5340 | 0.25 | 0.3273 |
| MYL1 | 4632 | 1.14 | 0.3277 |
| ALDOA | 226 | 1.71 | 0.3278 |
| FHL1 | 2273 | -0.72 | 0.3292 |
| PDIA4 | 9601 | 1.50 | 0.3293 |
| SFN | 2810 | -2.41 | 0.3304 |
| DMD | 1756 | -0.95 | 0.3306 |
| VASP | 7408 | -1.01 | 0.3308 |
| MYH1 | 4619 | -1.18 | 0.3316 |
| STXBP2 | 6813 | 1.17 | 0.3316 |
| MYL3 | 4634 | 1.12 | 0.3316 |
| F2 | 2147 | 1.46 | 0.3317 |
| LCP1 | 3936 | 1.11 | 0.3328 |
| TMED10 | 10972 | -1.34 | 0.3341 |
| SERPINA6 | 866 | -1.52 | 0.3351 |
| VPS13D | 55187 | 2.24 | 0.3354 |
| ATP6 | 4508 | 0.02 | 0.3356 |
| CYFIP2 | 26999 | -0.09 | 0.3356 |
| ATP4A | 495 | 0.24 | 0.3356 |
| INF2 | 64423 | 0.27 | 0.3356 |
| TKT | 7086 | 0.29 | 0.3356 |
| KIF5B | 3799 | -0.38 | 0.3356 |
| ICAM5 | 7087 | -0.63 | 0.3356 |
| SH3D19 | 152503 | 0.39 | 0.3356 |
| STT3A | 3703 | -0.42 | 0.3356 |
| LRRFIP1 | 9208 | -0.31 | 0.3356 |
| ENDOD1 | 23052 | 0.14 | 0.3356 |
| TMED7-TICAM2 | 100302736 | 0.39 | 0.3356 |
| TOR4A | 54863 | 0.37 | 0.3356 |
| TCP1 | 6950 | 0.34 | 0.3356 |
| CUL4B | 8450 | 0.32 | 0.3356 |
| SPERT | 220082 | 0.65 | 0.3356 |
| SYTL4 | 94121 | -0.51 | 0.3356 |
| DNM1L | 10059 | -0.57 | 0.3356 |
| HNRNPA0 | 10949 | -0.59 | 0.3356 |
| HMHA1 | 23526 | -0.26 | 0.3356 |
| KRT6C | 286887 | 0.62 | 0.3356 |
| APOF | 319 | -0.47 | 0.3356 |
| KRT6A | 3853 | 0.62 | 0.3356 |
| ARHGDIB | 397 | -0.57 | 0.3356 |
| SERPINB1 | 1992 | 0.42 | 0.3356 |
| RAB27A | 5873 | 0.58 | 0.3356 |
| ORMDL1 | 94101 | 0.61 | 0.3356 |
| CIT | 11113 | -0.39 | 0.3356 |
| CFHR3 | 10878 | 0.72 | 0.3356 |
| ITPR1 | 3708 | 0.34 | 0.3356 |
| KRT14 | 3861 | 0.60 | 0.3356 |
| RAP2C | 57826 | 0.56 | 0.3356 |
| TUBB2A | 7280 | 0.66 | 0.3356 |
| C6orf25 | 80739 | 0.71 | 0.3356 |
| CNDP1 | 84735 | 0.47 | 0.3356 |
| NIPSNAP1 | 8508 | 1.07 | 0.3356 |
| PRKAR1A | 5573 | 0.49 | 0.3356 |
| ATP1A2 | 477 | 0.24 | 0.3356 |
| MYLK | 4638 | 0.34 | 0.3356 |
| SPTBN1 | 6711 | 0.22 | 0.3356 |
| H1F0 | 3005 | -0.60 | 0.3356 |
| ALOX5AP | 241 | -0.68 | 0.3356 |
| ELOVL7 | 79993 | -0.58 | 0.3356 |
| NES | 10763 | -0.53 | 0.3356 |
| SLC2A14 | 144195 | -0.70 | 0.3356 |
| F13B | 2165 | -0.72 | 0.3356 |
| HIST1H2BA | 255626 | -0.86 | 0.3356 |
| GNAZ | 2781 | -0.32 | 0.3356 |
| GOT2 | 2806 | -0.58 | 0.3356 |
| TMED5 | 50999 | 0.63 | 0.3356 |
| NRDE2 | 55051 | -0.93 | 0.3356 |
| AIM1L | 55057 | -0.49 | 0.3356 |
| TMEM30A | 55754 | -0.61 | 0.3356 |
| EIF5A2 | 56648 | -0.60 | 0.3356 |
| RAB5A | 5868 | 0.53 | 0.3356 |
| SHMT2 | 6472 | -0.36 | 0.3356 |
| ADIPOQ | 9370 | 0.55 | 0.3356 |
| PRDX6 | 9588 | -0.62 | 0.3356 |
| KIAA0753 | 9851 | -0.55 | 0.3356 |
| ECE1 | 1889 | 0.52 | 0.3356 |
| FAU | 2197 | 0.72 | 0.3356 |
| ORMDL2 | 29095 | 0.60 | 0.3356 |
| RPL19 | 6143 | 0.69 | 0.3356 |
| ALDOC | 230 | 0.60 | 0.3356 |
| KRT16 | 3868 | 0.72 | 0.3356 |
| PCBP4 | 57060 | 0.49 | 0.3356 |
| HNRNPA1 | 3178 | -0.39 | 0.3356 |
| NFATC3 | 4775 | -0.65 | 0.3356 |
| RPS16 | 6217 | -0.83 | 0.3356 |
| UTS2 | 10911 | -0.79 | 0.3356 |
| COX17 | 10063 | 0.58 | 0.3356 |
| COPE | 11316 | 0.53 | 0.3356 |
| ENO3 | 2027 | 0.51 | 0.3356 |
| FKBP1A | 2280 | 0.74 | 0.3356 |
| GFAP | 2670 | 0.57 | 0.3356 |
| GNAS | 2778 | 0.39 | 0.3356 |
| TUBB2B | 347733 | 0.65 | 0.3356 |
| UBE2NL | 389898 | 0.51 | 0.3356 |
| NDUFB9 | 4715 | 0.64 | 0.3356 |
| TNFRSF11B | 4982 | 0.49 | 0.3356 |
| CYCS | 54205 | 0.68 | 0.3356 |
| UGGT1 | 56886 | 0.35 | 0.3356 |
| RAP2A | 5911 | 0.55 | 0.3356 |
| RAP2B | 5912 | 0.55 | 0.3356 |
| SH3BGRL | 6451 | 0.59 | 0.3356 |
| SUMO2 | 6613 | 0.57 | 0.3356 |
| UBE2N | 7334 | 0.53 | 0.3356 |
| MAP7D3 | 79649 | 0.47 | 0.3356 |
| RNGTT | 8732 | 0.64 | 0.3356 |
| LMNA | 4000 | 0.43 | 0.3356 |
| PGAM5 | 192111 | 0.55 | 0.3356 |
| LBP | 3929 | -0.50 | 0.3356 |
| IARS2 | 55699 | 0.13 | 0.3356 |
| RAB5B | 5869 | 0.54 | 0.3356 |
| PITRM1 | 10531 | -0.34 | 0.3356 |
| TUBA3E | 112714 | -0.50 | 0.3356 |
| TUBA3D | 113457 | -0.50 | 0.3356 |
| COX6B1 | 1340 | -0.70 | 0.3356 |
| RAB21 | 23011 | -0.46 | 0.3356 |
| DPY19L1 | 23333 | -0.30 | 0.3356 |
| KRT18 | 3875 | -0.39 | 0.3356 |
| NME1 | 4830 | -0.52 | 0.3356 |
| DNAJB11 | 51726 | -0.48 | 0.3356 |
| MBD5 | 55777 | -0.61 | 0.3356 |
| APOM | 55937 | -0.58 | 0.3356 |
| VAMP1 | 6843 | -0.63 | 0.3356 |
| TUBA3C | 7278 | -0.50 | 0.3356 |
| MCU | 90550 | -0.73 | 0.3356 |
| MTDH | 92140 | 0.54 | 0.3356 |
| HSP90AB1 | 3326 | -0.64 | 0.3356 |
| RPS3A | 6189 | -0.65 | 0.3356 |
| H2AFY2 | 55506 | -0.54 | 0.3356 |
| DCP2 | 167227 | -0.73 | 0.3356 |
| PRDX3 | 10935 | 0.47 | 0.3356 |
| SLC27A3 | 11000 | 0.37 | 0.3356 |
| DYNLL2 | 140735 | 0.55 | 0.3356 |
| ALOX12 | 239 | 0.40 | 0.3356 |
| ATP2C1 | 27032 | 0.33 | 0.3356 |
| GNAI3 | 2773 | 0.41 | 0.3356 |
| LTF | 4057 | 0.66 | 0.3356 |
| NDUFS4 | 4724 | 0.60 | 0.3356 |
| TMED7 | 51014 | 0.43 | 0.3356 |
| PCOLCE | 5118 | 0.58 | 0.3356 |
| MAP7D1 | 55700 | 0.44 | 0.3356 |
| AZGP1 | 563 | 0.56 | 0.3356 |
| AKR7A2 | 8574 | 0.48 | 0.3356 |
| DYNLL1 | 8655 | 0.55 | 0.3356 |
| BANF1 | 8815 | 0.64 | 0.3356 |
| MAP4K4 | 9448 | 0.51 | 0.3356 |
| N4BP1 | 9683 | 0.82 | 0.3356 |
| SAMHD1 | 25939 | 0.54 | 0.3356 |
| IFI16 | 3428 | 0.38 | 0.3356 |
| S100A6 | 6277 | 0.73 | 0.3356 |
| GRPEL1 | 80273 | 0.38 | 0.3356 |
| AK7 | 122481 | -0.53 | 0.3356 |
| VPS13B | 157680 | -0.56 | 0.3356 |
| HSD17B4 | 3295 | -0.57 | 0.3356 |
| HSPA2 | 3306 | -0.34 | 0.3356 |
| NME2 | 4831 | -0.55 | 0.3356 |
| CYB5R1 | 51706 | -0.55 | 0.3356 |
| NME1-NME2 | 654364 | -0.46 | 0.3356 |
| TNNC2 | 7125 | -0.51 | 0.3356 |
| CAPNS1 | 826 | -0.50 | 0.3356 |
| CD5L | 922 | -0.72 | 0.3356 |
| NDUFA9 | 4704 | -0.41 | 0.3356 |
| PRG4 | 10216 | 0.41 | 0.3356 |
| CPA3 | 1359 | 0.71 | 0.3356 |
| TOR1AIP1 | 26092 | 0.40 | 0.3356 |
| GNAI1 | 2770 | 0.42 | 0.3356 |
| GNAL | 2774 | 0.43 | 0.3356 |
| EYS | 346007 | 0.44 | 0.3356 |
| KPNA2 | 3838 | 0.69 | 0.3356 |
| SUMO4 | 387082 | 0.55 | 0.3356 |
| RPL7 | 6129 | 0.42 | 0.3356 |
| SUMO3 | 6612 | 0.55 | 0.3356 |
| MAP7 | 9053 | 0.45 | 0.3356 |
| MYO18A | 399687 | 0.17 | 0.3356 |
| F11 | 2160 | -0.35 | 0.3356 |
| MKI67 | 4288 | -0.42 | 0.3356 |
| GNAO1 | 2775 | 0.41 | 0.3356 |
| GNAT1 | 2779 | 0.41 | 0.3356 |
| GNAT2 | 2780 | 0.40 | 0.3356 |
| GNAT3 | 346562 | 0.41 | 0.3356 |
| SNAP29 | 9342 | 0.38 | 0.3356 |
| GAL3ST1 | 9514 | 0.63 | 0.3356 |
| SPCS1 | 28972 | -0.10 | 0.3356 |
| TMPO | 7112 | -0.07 | 0.3356 |
| HNRNPU | 3192 | 0.07 | 0.3356 |
| RPL3 | 6122 | 0.11 | 0.3356 |
| EIF5A | 1984 | 0.04 | 0.3356 |
| SDHB | 6390 | -0.01 | 0.3356 |
| TAPBP | 6892 | -0.96 | 0.3366 |
| GSTO1 | 9446 | 1.51 | 0.3387 |
| DPM3 | 54344 | 0.64 | 0.3397 |
| OGDH | 4967 | -0.76 | 0.3401 |
| ATP5F1 | 515 | 1.60 | 0.3416 |
| MMRN1 | 22915 | 0.93 | 0.3422 |
| MYH2 | 4620 | -1.05 | 0.3434 |
| APOC4 | 346 | -1.54 | 0.3445 |
| SERPINA7 | 6906 | 1.25 | 0.3449 |
| AK3 | 50808 | -1.34 | 0.3478 |
| LIMS3L | 100288695 | -0.51 | 0.3479 |
| PTPRJ | 5795 | 1.31 | 0.3480 |
| ILK | 3611 | -1.50 | 0.3490 |
| MYL6 | 4637 | 1.27 | 0.3493 |
| APP | 351 | 1.18 | 0.3514 |
| RPL35 | 11224 | -0.69 | 0.3529 |
| CYB5R3 | 1727 | 1.66 | 0.3542 |
| BIN2 | 51411 | 1.44 | 0.3542 |
| TUBB3 | 10381 | -1.84 | 0.3553 |
| LRG1 | 116844 | -0.67 | 0.3555 |
| SERPINA1 | 5265 | -0.88 | 0.3560 |
| C8A | 731 | 0.98 | 0.3574 |
| C3 | 718 | 0.17 | 0.3586 |
| SACM1L | 22908 | -1.23 | 0.3630 |
| CORO1C | 23603 | 1.21 | 0.3661 |
| ARPC1B | 10095 | -1.88 | 0.3662 |
| RPL18 | 6141 | -0.59 | 0.3694 |
| METTL7A | 25840 | -0.48 | 0.3707 |
| C4BPA | 722 | -1.52 | 0.3737 |
| RPS4Y1 | 6192 | -1.03 | 0.3754 |
| ESAM | 90952 | -1.10 | 0.3756 |
| TGOLN2 | 10618 | -0.85 | 0.3763 |
| MYL12B | 103910 | -0.77 | 0.3766 |
| ANO6 | 196527 | -0.65 | 0.3779 |
| TMED9 | 54732 | -1.21 | 0.3779 |
| ACADM | 34 | -0.49 | 0.3780 |
| MYL12A | 10627 | -0.76 | 0.3787 |
| RPS4Y2 | 140032 | -0.99 | 0.3793 |
| ATL3 | 25923 | -1.07 | 0.3797 |
| C22orf23 | 84645 | -3.07 | 0.3821 |
| VTN | 7448 | 1.03 | 0.3838 |
| YWHAZ | 7534 | 1.03 | 0.3852 |
| IGFALS | 3483 | -0.40 | 0.3856 |
| A1BG | 1 | 0.81 | 0.3874 |
| SELP | 6403 | 1.81 | 0.3876 |
| CALM2 | 805 | -1.35 | 0.3920 |
| CALM1 | 801 | -1.36 | 0.3926 |
| CALM3 | 808 | -1.38 | 0.3943 |
| ACO2 | 50 | 1.30 | 0.3954 |
| MYH3 | 4621 | -0.63 | 0.3979 |
| EEF1A2 | 1917 | 0.99 | 0.3990 |
| HIST2H2AB | 317772 | 1.82 | 0.4020 |
| PECAM1 | 5175 | 1.02 | 0.4042 |
| CFL1 | 1072 | 0.86 | 0.4048 |
| HABP2 | 3026 | 1.30 | 0.4103 |
| STOM | 2040 | 1.08 | 0.4104 |
| ITGB3 | 3690 | 1.01 | 0.4110 |
| PCBP1 | 5093 | 0.72 | 0.4140 |
| ATP5D | 513 | -1.41 | 0.4163 |
| C9 | 735 | -0.91 | 0.4175 |
| LETM1 | 3954 | -0.56 | 0.4179 |
| LPA | 4018 | -1.10 | 0.4186 |
| LMNB1 | 4001 | 1.15 | 0.4219 |
| EWSR1 | 2130 | 0.41 | 0.4221 |
| HBD | 3045 | -1.10 | 0.4242 |
| SFPQ | 6421 | -1.06 | 0.4255 |
| SEC22B | 9554 | 1.31 | 0.4309 |
| DCTN1 | 1639 | 0.27 | 0.4315 |
| ATP5O | 539 | -1.04 | 0.4330 |
| SDHA | 6389 | 0.84 | 0.4361 |
| HNRNPM | 4670 | 0.98 | 0.4378 |
| RPS2 | 6187 | -1.07 | 0.4393 |
| APOC3 | 345 | 1.02 | 0.4457 |
| UQCRC1 | 7384 | -0.85 | 0.4491 |
| DHRS7 | 51635 | -1.24 | 0.4507 |
| APOC2 | 344 | 1.13 | 0.4521 |
| TLN1 | 7094 | 0.26 | 0.4554 |
| HSPD1 | 3329 | 0.76 | 0.4555 |
| APOE | 348 | 0.86 | 0.4575 |
| RAB5C | 5878 | 0.93 | 0.4603 |
| GNAI2 | 2771 | 1.40 | 0.4637 |
| SERPING1 | 710 | 1.33 | 0.4668 |
| NNT | 23530 | -1.02 | 0.4679 |
| PPIB | 5479 | 1.32 | 0.4714 |
| SLC25A5 | 292 | -1.12 | 0.4725 |
| HBA1 | 3039 | -0.44 | 0.4735 |
| HBA2 | 3040 | -0.44 | 0.4735 |
| SMIM1 | 388588 | -0.84 | 0.4755 |
| ARHGAP35 | 2909 | 1.19 | 0.4769 |
| HRG | 3273 | -1.08 | 0.4777 |
| HNRNPA3 | 220988 | 0.88 | 0.4805 |
| STX7 | 8417 | 0.77 | 0.4811 |
| GPX1 | 2876 | 1.44 | 0.4811 |
| CYB5B | 80777 | -1.30 | 0.4862 |
| TMEM109 | 79073 | -1.30 | 0.4890 |
| ATP5J2-PTCD1 | 100526740 | -0.79 | 0.4895 |
| CLTC | 1213 | 0.55 | 0.4903 |
| 44446 | 989 | 0.59 | 0.4928 |
| SFXN3 | 81855 | 0.65 | 0.4958 |
| PLEC | 5339 | 0.25 | 0.4997 |
| SERPINF1 | 5176 | 0.96 | 0.5013 |
| CD84 | 8832 | 1.10 | 0.5041 |
| VCP | 7415 | 0.60 | 0.5054 |
| ACTA1 | 58 | 1.70 | 0.5061 |
| PLEK | 5341 | -1.14 | 0.5077 |
| ITGA6 | 3655 | 0.79 | 0.5088 |
| HK1 | 3098 | -0.48 | 0.5089 |
| PFN1 | 5216 | -0.23 | 0.5104 |
| CD36 | 948 | -0.78 | 0.5111 |
| CAPZA1 | 829 | 0.63 | 0.5123 |
| ATP2A3 | 489 | 0.68 | 0.5133 |
| NDUFS1 | 4719 | -0.53 | 0.5163 |
| VAMP2 | 6844 | -1.13 | 0.5175 |
| VAMP3 | 9341 | -1.09 | 0.5198 |
| TAGLN2 | 8407 | 1.00 | 0.5200 |
| TAP1 | 6890 | 0.75 | 0.5222 |
| UQCRB | 7381 | 0.90 | 0.5231 |
| CERS2 | 29956 | -0.94 | 0.5237 |
| SLC25A3 | 5250 | -1.40 | 0.5270 |
| CD226 | 10666 | 1.13 | 0.5280 |
| GNB4 | 59345 | 0.73 | 0.5286 |
| CALR | 811 | 1.01 | 0.5290 |
| GNB2 | 2783 | 0.74 | 0.5301 |
| PZP | 5858 | -0.62 | 0.5321 |
| GRB2 | 2885 | -0.64 | 0.5325 |
| ATP5L | 10632 | 1.71 | 0.5335 |
| TPI1 | 7167 | 1.08 | 0.5351 |
| SAA1 | 6288 | -0.91 | 0.5368 |
| PRKCB | 5579 | 0.57 | 0.5373 |
| SCAMP2 | 10066 | 0.75 | 0.5378 |
| YBX1 | 4904 | 0.80 | 0.5392 |
| ATP5J2 | 9551 | -1.02 | 0.5398 |
| SLC25A11 | 8402 | 0.81 | 0.5398 |
| YWHAE | 7531 | -1.36 | 0.5418 |
| AK2 | 204 | 0.78 | 0.5450 |
| EEF1B2 | 1933 | 0.56 | 0.5476 |
| CD9 | 928 | 0.86 | 0.5487 |
| CANX | 821 | 0.78 | 0.5523 |
| HPR | 3250 | -0.93 | 0.5526 |
| WTH3DI | 150786 | 0.76 | 0.5529 |
| RAB6C | 84084 | 0.76 | 0.5529 |
| RPS4X | 6191 | -0.64 | 0.5531 |
| F11R | 50848 | 1.17 | 0.5547 |
| CFHR1 | 3078 | 0.97 | 0.5547 |
| ETFA | 2108 | 0.90 | 0.5577 |
| WDR1 | 9948 | -0.60 | 0.5585 |
| TPM4 | 7171 | 0.39 | 0.5586 |
| H3F3C | 440093 | 1.12 | 0.5599 |
| STOML3 | 161003 | -0.90 | 0.5614 |
| HNRNPH1 | 3187 | 0.47 | 0.5620 |
| HMGB1 | 3146 | -1.41 | 0.5639 |
| FH | 2271 | -0.58 | 0.5678 |
| ESYT1 | 23344 | 0.57 | 0.5692 |
| F13A1 | 2162 | 0.59 | 0.5700 |
| TAOK3 | 51347 | -0.49 | 0.5701 |
| TOMM22 | 56993 | 0.60 | 0.5704 |
| APOA2 | 336 | -0.74 | 0.5717 |
| IDH2 | 3418 | -0.98 | 0.5736 |
| MLEC | 9761 | -0.92 | 0.5757 |
| HIST1H2BB | 3018 | 1.54 | 0.5760 |
| HIST1H2BO | 8348 | 1.54 | 0.5760 |
| HIST2H2BE | 8349 | 1.54 | 0.5760 |
| HIST1H2BJ | 8970 | 1.54 | 0.5760 |
| CA1 | 759 | -0.57 | 0.5778 |
| ACTR3 | 10096 | -1.02 | 0.5798 |
| FLOT1 | 10211 | 0.44 | 0.5805 |
| ACTR2 | 10097 | -0.91 | 0.5829 |
| CD44 | 960 | 0.50 | 0.5853 |
| MDH2 | 4191 | 0.90 | 0.5868 |
| ATP5J | 522 | 0.81 | 0.5985 |
| RAB32 | 10981 | 0.75 | 0.6006 |
| GC | 2638 | 0.89 | 0.6012 |
| STX11 | 8676 | -0.84 | 0.6022 |
| ITGB1 | 3688 | 0.72 | 0.6031 |
| TREML1 | 340205 | 1.01 | 0.6041 |
| CYFIP1 | 23191 | 0.12 | 0.6046 |
| UQCR10 | 29796 | 0.67 | 0.6076 |
| FGB | 2244 | 0.08 | 0.6077 |
| RPS6 | 6194 | -0.63 | 0.6079 |
| FLNC | 2318 | -0.75 | 0.6108 |
| CAPZA2 | 830 | -0.80 | 0.6110 |
| SEPP1 | 6414 | -0.70 | 0.6128 |
| FLNB | 2317 | 0.40 | 0.6147 |
| HIST2H3PS2 | 440686 | 1.11 | 0.6147 |
| VCL | 7414 | 0.60 | 0.6148 |
| IMMT | 10989 | 0.89 | 0.6154 |
| TMED4 | 222068 | -0.55 | 0.6165 |
| ZYX | 7791 | 0.76 | 0.6201 |
| ARPC4-TTLL3 | 100526693 | -0.49 | 0.6222 |
| CST7 | 8530 | -1.51 | 0.6232 |
| AFM | 173 | -0.49 | 0.6266 |
| RPL28 | 6158 | 0.75 | 0.6274 |
| CD74 | 972 | -0.01 | 0.6278 |
| MYL9 | 10398 | 1.38 | 0.6281 |
| VWF | 7450 | 0.60 | 0.6283 |
| RPN2 | 6185 | -0.70 | 0.6287 |
| FBLN1 | 2192 | 0.94 | 0.6303 |
| ATP5B | 506 | 0.50 | 0.6307 |
| GNA12 | 2768 | 0.54 | 0.6319 |
| GNA13 | 10672 | 0.53 | 0.6322 |
| C6 | 729 | 0.54 | 0.6368 |
| STIM1 | 6786 | -0.43 | 0.6395 |
| RAB14 | 51552 | -0.82 | 0.6397 |
| RAB27B | 5874 | 0.60 | 0.6417 |
| LUM | 4060 | -0.98 | 0.6480 |
| HSPE1 | 3336 | 0.95 | 0.6497 |
| SCCPDH | 51097 | -0.40 | 0.6526 |
| ROCK2 | 9475 | 0.47 | 0.6560 |
| HADHA | 3030 | -0.59 | 0.6566 |
| PARVB | 29780 | -0.87 | 0.6642 |
| GPI | 2821 | 0.45 | 0.6703 |
| HBB | 3043 | 0.53 | 0.6740 |
| C7 | 730 | -0.56 | 0.6752 |
| VAPA | 9218 | -0.46 | 0.6785 |
| GNB1 | 2782 | -0.76 | 0.6789 |
| HMGB2 | 3148 | -0.58 | 0.6810 |
| PRDX5 | 25824 | 0.68 | 0.6817 |
| RSU1 | 6251 | -0.71 | 0.6819 |
| PDIA6 | 10130 | -0.69 | 0.6820 |
| ARPC4 | 10093 | -1.01 | 0.6823 |
| RAB1A | 5861 | -0.67 | 0.6827 |
| TUBA1C | 84790 | 0.55 | 0.6830 |
| TUBA1B | 10376 | 0.55 | 0.6852 |
| TUBA1A | 7846 | 0.55 | 0.6858 |
| CPT1A | 1374 | -0.63 | 0.6875 |
| HIST3H2BB | 128312 | -0.77 | 0.6882 |
| B2M | 567 | 0.91 | 0.6898 |
| ACTN1 | 87 | 0.47 | 0.6919 |
| HYOU1 | 10525 | 0.49 | 0.6948 |
| SLC4A1 | 6521 | -0.37 | 0.6962 |
| TGFB1 | 7040 | -0.44 | 0.6991 |
| DCD | 117159 | 0.47 | 0.7009 |
| EZR | 7430 | -0.35 | 0.7013 |
| C1QBP | 708 | -0.71 | 0.7014 |
| HIST1H2AE | 3012 | 0.85 | 0.7040 |
| HIST1H2AD | 3013 | 0.85 | 0.7040 |
| H2AFJ | 55766 | 0.85 | 0.7040 |
| HIST2H2AA4 | 723790 | 0.85 | 0.7040 |
| HIST1H2AI | 8329 | 0.85 | 0.7040 |
| HIST1H2AK | 8330 | 0.85 | 0.7040 |
| HIST1H2AJ | 8331 | 0.85 | 0.7040 |
| HIST1H2AL | 8332 | 0.85 | 0.7040 |
| HIST1H2AC | 8334 | 0.85 | 0.7040 |
| HIST1H2AB | 8335 | 0.85 | 0.7040 |
| HIST1H2AM | 8336 | 0.85 | 0.7040 |
| HIST2H2AA3 | 8337 | 0.85 | 0.7040 |
| HIST2H2AC | 8338 | 0.85 | 0.7040 |
| HIST1H2AH | 85235 | 0.85 | 0.7040 |
| HIST1H2AG | 8969 | 0.85 | 0.7040 |
| HIST3H2A | 92815 | 0.85 | 0.7040 |
| HLA-C | 3107 | 0.86 | 0.7081 |
| ATP5I | 521 | -0.99 | 0.7087 |
| C8B | 732 | 0.51 | 0.7099 |
| LRRC59 | 55379 | -0.54 | 0.7131 |
| PGRMC1 | 10857 | 0.69 | 0.7136 |
| EHD3 | 30845 | 0.33 | 0.7147 |
| GSN | 2934 | 0.12 | 0.7163 |
| LGALS3BP | 3959 | 0.42 | 0.7174 |
| RDX | 5962 | -0.35 | 0.7185 |
| EBF2 | 64641 | 0.44 | 0.7199 |
| GNB3 | 2784 | -0.41 | 0.7214 |
| MAOA | 4128 | -0.22 | 0.7232 |
| GPD2 | 2820 | -0.60 | 0.7239 |
| GSTP1 | 2950 | -0.45 | 0.7243 |
| ECM1 | 1893 | 0.48 | 0.7251 |
| APMAP | 57136 | -0.42 | 0.7277 |
| DBN1 | 1627 | 0.73 | 0.7302 |
| MYH9 | 4627 | -0.15 | 0.7313 |
| KRT2 | 3849 | -0.41 | 0.7318 |
| ATP5A1 | 498 | -0.13 | 0.7342 |
| F5 | 2153 | 0.26 | 0.7366 |
| GP1BB | 2812 | -0.79 | 0.7387 |
| TAP2 | 6891 | -0.36 | 0.7396 |
| COX5B | 1329 | -0.66 | 0.7401 |
| PRKCSH | 5589 | 0.45 | 0.7408 |
| ORM2 | 5005 | -0.54 | 0.7443 |
| H3F3A | 3020 | 0.62 | 0.7449 |
| H3F3B | 3021 | 0.62 | 0.7449 |
| HERC2 | 8924 | -0.29 | 0.7450 |
| TPM2 | 7169 | 0.71 | 0.7474 |
| RPS14 | 6208 | 0.50 | 0.7483 |
| H1FX | 8971 | -0.26 | 0.7498 |
| ATP2A2 | 488 | 0.29 | 0.7522 |
| KLKB1 | 3818 | 0.39 | 0.7594 |
| APOH | 350 | -0.62 | 0.7600 |
| RAB1B | 81876 | 0.51 | 0.7603 |
| GNAQ | 2776 | -0.55 | 0.7608 |
| RER1 | 11079 | 0.53 | 0.7609 |
| RPL17 | 6139 | -0.36 | 0.7614 |
| LYN | 4067 | -0.45 | 0.7657 |
| ETFB | 2109 | 0.47 | 0.7667 |
| RPL17-C18orf32 | 100526842 | -0.32 | 0.7718 |
| ADAM10 | 102 | 0.46 | 0.7733 |
| C1QB | 713 | 0.56 | 0.7753 |
| KIF11 | 3832 | -0.34 | 0.7771 |
| PGK1 | 5230 | 0.31 | 0.7772 |
| C1RL | 51279 | -0.36 | 0.7810 |
| MYH7B | 57644 | 0.06 | 0.7847 |
| HLA-A | 3105 | -0.51 | 0.7852 |
| TBXAS1 | 6916 | 0.40 | 0.7852 |
| ENO1 | 2023 | -0.32 | 0.7875 |
| MYH8 | 4626 | 0.06 | 0.7912 |
| MAPRE2 | 10982 | -0.40 | 0.7914 |
| LGALSL | 29094 | -0.37 | 0.7955 |
| GAPDH | 2597 | 0.40 | 0.7960 |
| LOC110384692 | 110384692 | -0.52 | 0.7997 |
| C1QA | 712 | -0.57 | 0.8006 |
| MSN | 4478 | -0.25 | 0.8014 |
| CHCHD3 | 54927 | 0.37 | 0.8055 |
| BCAP31 | 10134 | 0.53 | 0.8070 |
| TMOD3 | 29766 | -0.40 | 0.8084 |
| HIST2H3C | 126961 | 0.39 | 0.8119 |
| HIST2H3A | 333932 | 0.39 | 0.8119 |
| HIST2H3D | 653604 | 0.39 | 0.8119 |
| HIST1H3A | 8350 | 0.39 | 0.8119 |
| HIST1H3D | 8351 | 0.39 | 0.8119 |
| HIST1H3C | 8352 | 0.39 | 0.8119 |
| HIST1H3E | 8353 | 0.39 | 0.8119 |
| HIST1H3I | 8354 | 0.39 | 0.8119 |
| HIST1H3G | 8355 | 0.39 | 0.8119 |
| HIST1H3J | 8356 | 0.39 | 0.8119 |
| HIST1H3H | 8357 | 0.39 | 0.8119 |
| HIST1H3B | 8358 | 0.39 | 0.8119 |
| HIST1H3F | 8968 | 0.39 | 0.8119 |
| HIST3H3 | 8290 | 0.39 | 0.8119 |
| FN1 | 2335 | 0.05 | 0.8122 |
| C4A | 720 | 0.34 | 0.8134 |
| MYL10 | 93408 | -0.38 | 0.8150 |
| ERAP1 | 51752 | -0.17 | 0.8196 |
| C2 | 717 | 0.24 | 0.8225 |
| HMGCL | 3155 | -0.17 | 0.8235 |
| SAA2 | 6289 | -0.34 | 0.8246 |
| LRBA | 987 | 0.15 | 0.8261 |
| KRT6B | 3854 | 0.18 | 0.8275 |
| UQCRC2 | 7385 | 0.32 | 0.8278 |
| FERMT3 | 83706 | 0.40 | 0.8343 |
| SPARC | 6678 | -0.34 | 0.8365 |
| CORO1A | 11151 | 0.33 | 0.8407 |
| KRT10 | 3858 | -0.27 | 0.8431 |
| MYH14 | 79784 | 0.17 | 0.8473 |
| FETUB | 26998 | 0.11 | 0.8493 |
| ARL6IP5 | 10550 | -0.33 | 0.8499 |
| NID1 | 4811 | 0.20 | 0.8528 |
| IDH3A | 3419 | -0.21 | 0.8543 |
| GLUD1 | 2746 | 0.30 | 0.8550 |
| GANAB | 23193 | 0.24 | 0.8562 |
| CAPZB | 832 | -0.29 | 0.8594 |
| ARL8B | 55207 | 0.14 | 0.8594 |
| VIM | 7431 | 0.06 | 0.8621 |
| ITIH3 | 3699 | -0.20 | 0.8634 |
| RPS13 | 6207 | 0.25 | 0.8637 |
| ME2 | 4200 | -0.11 | 0.8657 |
| HSD17B10 | 3028 | 0.19 | 0.8665 |
| C21orf33 | 8209 | 0.25 | 0.8674 |
| LOC102724023 | 102724023 | 0.25 | 0.8680 |
| EMILIN1 | 11117 | 0.22 | 0.8685 |
| NDUFB11 | 54539 | -0.19 | 0.8702 |
| PRDX1 | 5052 | 0.14 | 0.8734 |
| IER3IP1 | 51124 | 0.20 | 0.8744 |
| RPS18 | 6222 | 0.19 | 0.8755 |
| APOOL | 139322 | 0.23 | 0.8760 |
| CMTM6 | 54918 | 0.21 | 0.8764 |
| HSP90AA1 | 3320 | 0.23 | 0.8780 |
| PGRMC2 | 10424 | -0.28 | 0.8791 |
| MNDA | 4332 | 0.22 | 0.8823 |
| HSPA9 | 3313 | -0.20 | 0.8855 |
| SAA2-SAA4 | 100528017 | 0.23 | 0.8857 |
| SPCS2 | 9789 | 0.19 | 0.8871 |
| CD99 | 4267 | 0.12 | 0.8881 |
| HNRNPK | 3190 | 0.09 | 0.8888 |
| HIST1H1D | 3007 | -0.24 | 0.8898 |
| ACTC1 | 70 | 0.41 | 0.8925 |
| 110599563 | 110599563 | 0.10 | 0.8931 |
| LY6G6F | 259215 | 0.10 | 0.8960 |
| APOA1 | 335 | 0.05 | 0.8967 |
| KNG1 | 3827 | -0.17 | 0.9045 |
| DDOST | 1650 | 0.09 | 0.9099 |
| PHB | 5245 | 0.19 | 0.9118 |
| CS | 1431 | 0.16 | 0.9131 |
| GLS | 2744 | 0.13 | 0.9136 |
| COPA | 1314 | 0.10 | 0.9143 |
| THBS1 | 7057 | -0.04 | 0.9180 |
| HSPA6 | 3310 | -0.11 | 0.9200 |
| NAP1L1 | 4673 | -0.21 | 0.9205 |
| ANXA6 | 309 | 0.11 | 0.9245 |
| TRIM33 | 51592 | 0.10 | 0.9253 |
| NRGN | 4900 | 0.13 | 0.9286 |
| CDS2 | 8760 | 0.15 | 0.9297 |
| PTGS1 | 5742 | 0.15 | 0.9349 |
| SSR4 | 6748 | -0.11 | 0.9377 |
| AIFM1 | 9131 | -0.05 | 0.9411 |
| ACTN4 | 81 | 0.13 | 0.9419 |
| RAB2A | 5862 | -0.05 | 0.9442 |
| MTPN | 136319 | 0.10 | 0.9455 |
| CTTN | 2017 | 0.05 | 0.9458 |
| SH3BGRL3 | 83442 | 0.16 | 0.9464 |
| ERP44 | 23071 | -0.05 | 0.9466 |
| ITGA2B | 3674 | -0.03 | 0.9478 |
| HSD17B12 | 51144 | 0.09 | 0.9484 |
| EEF1A1 | 1915 | -0.08 | 0.9490 |
| STOML2 | 30968 | -0.04 | 0.9501 |
| RPS11 | 6205 | 0.06 | 0.9520 |
| ACAT1 | 38 | 0.08 | 0.9525 |
| NDUFV1 | 4723 | 0.06 | 0.9534 |
| STAU1 | 6780 | 0.06 | 0.9537 |
| STAU2 | 27067 | 0.06 | 0.9542 |
| HIST1H2BL | 8340 | 0.09 | 0.9544 |
| LDHA | 3939 | 0.09 | 0.9548 |
| CNN2 | 1265 | 0.04 | 0.9558 |
| CNN3 | 1266 | 0.04 | 0.9559 |
| SLC25A6 | 293 | 0.09 | 0.9565 |
| P2RX1 | 5023 | 0.04 | 0.9597 |
| COX7C | 1350 | -0.06 | 0.9618 |
| ARF1 | 375 | -0.07 | 0.9635 |
| ARF3 | 377 | -0.07 | 0.9641 |
| AHNAK | 79026 | -0.02 | 0.9645 |
| UQCRFS1 | 7386 | -0.08 | 0.9645 |
| ARHGDIA | 396 | 0.04 | 0.9662 |
| LDHB | 3945 | -0.06 | 0.9676 |
| CD14 | 929 | 0.03 | 0.9701 |
| CMTM5 | 116173 | -0.07 | 0.9703 |
| PROS1 | 5627 | 0.07 | 0.9703 |
| SERPINA3 | 12 | -0.02 | 0.9712 |
| IQGAP1 | 8826 | 0.03 | 0.9716 |
| CLIC1 | 1192 | 0.06 | 0.9718 |
| HMGN1 | 3150 | 0.03 | 0.9723 |
| COX4I1 | 1327 | 0.04 | 0.9726 |
| PCBP3 | 54039 | 0.02 | 0.9740 |
| PCBP2 | 5094 | 0.02 | 0.9749 |
| RPS3 | 6188 | 0.04 | 0.9758 |
| ATPIF1 | 93974 | 0.03 | 0.9771 |
| RAB18 | 22931 | -0.02 | 0.9780 |
| CSRP1 | 1465 | 0.02 | 0.9782 |
| MRVI1 | 10335 | 0.02 | 0.9785 |
| CYB5A | 1528 | -0.02 | 0.9787 |
| CPN2 | 1370 | 0.02 | 0.9788 |
| PKM | 5315 | 0.04 | 0.9819 |
| SLMAP | 7871 | -0.02 | 0.9832 |
| RAP1B | 5908 | -0.03 | 0.9844 |
| HACD4 | 401494 | -0.01 | 0.9847 |
| TMSB10 | 9168 | -0.02 | 0.9849 |
| OPA1 | 4976 | -0.01 | 0.9861 |
| PHB2 | 11331 | 0.03 | 0.9865 |
| RAB11B | 9230 | 0.02 | 0.9883 |
| PLXDC2 | 84898 | -0.01 | 0.9885 |
| RAB11A | 8766 | 0.02 | 0.9887 |
| CALML3 | 810 | -0.02 | 0.9903 |
| CA2 | 760 | -0.01 | 0.9904 |
| C1QC | 714 | 0.02 | 0.9906 |
| ARPC2 | 10109 | -0.02 | 0.9919 |
| ACADVL | 37 | 0.01 | 0.9952 |
| CCDC168 | 643677 | 0.00 | 0.9954 |
| TSPO | 706 | -0.01 | 0.9962 |
| C8G | 733 | 0.00 | 0.9973 |
| USMG5 | 84833 | 0.00 | 0.9987 |
| KRT9 | 3857 | 0.00 | 0.9999 |
