# Supplementary material for: Dawn-to-dusk dry fasting induces anti-atherosclerotic, anti-inflammatory, and anti-tumorigenic proteome in peripheral blood mononuclear cells in subjects with metabolic syndrome
Source: Metabol Open. 2022 Nov 1;16:100214. doi: 10.1016/j.metop.2022.100214 (PMC9731888; doi:10.1016/j.metop.2022.100214)
Supplement: Supplementary Table S3 [file mmc3.docx]

| **Supplementary Table S3. Gene Protein Products (GP) Levels that Are Up- or Downregulated One Week after 4-Week Dawn-to-Dusk Dry Fasting (V3) Compared with the GP Levels at the End of 4-Week Dawn-to-Dusk Dry Fasting (V2)** | | | |
| --- | --- | --- | --- |
| **Gene Symbol** | **Gene ID** | **Average Paired Log2 Fold Change (V3/V2)** | **Paired P Value** |
| PGLYRP2 | 114770 | 2.26 | 0.0446 |
| SOD2 | 6648 | 4.17 | 0.0127 |
| ETFA | 2108 | 2.87 | 0.0290 |
| KRT77 | 374454 | 1.67 | 0.0447 |
| RAB1B | 81876 | 2.55 | 0.0478 |
| CFHR2 | 3080 | -3.08 | 0.0410 |
| ATP2A1 | 487 | -2.15 | 0.0336 |
| COX5B | 1329 | -2.94 | 0.0346 |
| LUM | 4060 | -3.61 | 0.0175 |
| PLEK | 5341 | -2.42 | 0.0466 |
| ATP5I | 521 | -3.91 | 0.0239 |
| F10 | 2159 | -2.96 | 0.0256 |
| GLUD2 | 2747 | -2.18 | 0.0385 |
| ANXA2 | 302 | 2.70 | 0.0536 |
| RTN3 | 10313 | 1.75 | 0.0827 |
| CD36 | 948 | 1.85 | 0.0827 |
| FLNC | 2318 | 1.62 | 0.0943 |
| MGST2 | 4258 | 2.01 | 0.0829 |
| H1F0 | 3005 | -1.88 | 0.0839 |
| TUBB4A | 10382 | -1.91 | 0.0826 |
| LIMS1 | 3987 | -2.17 | 0.0979 |
| ATP5C1 | 509 | -2.53 | 0.0946 |
| RPL27A | 6157 | -1.80 | 0.0834 |
| PLCG1 | 5335 | -2.64 | 0.0724 |
| PKM | 5315 | -2.04 | 0.0732 |
| LSP1 | 4046 | -2.34 | 0.0617 |
| LDHB | 3945 | -2.33 | 0.0848 |
| PDIA6 | 10130 | -2.59 | 0.0526 |
| MYL9 | 10398 | -3.54 | 0.0812 |
| F5 | 2153 | -0.39 | 0.0688 |
| HIST1H1C | 3006 | -0.49 | 0.0879 |
| VIM | 7431 | -0.49 | 0.0709 |
| APOH | 350 | 3.06 | 0.0707 |
| APOA4 | 337 | 0.67 | 0.0870 |
| RAB32 | 10981 | 1.94 | 0.1003 |
| COX7C | 1350 | -2.33 | 0.1004 |
| RPS4Y1 | 6192 | -1.77 | 0.1160 |
| RPS4Y2 | 140032 | -1.72 | 0.1164 |
| GNG5 | 2787 | -2.80 | 0.1187 |
| RAB1A | 5861 | 2.21 | 0.1200 |
| PGRMC1 | 10857 | -1.67 | 0.1219 |
| PPIB | 5479 | -1.57 | 0.1223 |
| GNB4 | 59345 | 1.24 | 0.1223 |
| GNB2 | 2783 | 1.26 | 0.1226 |
| SRPRB | 58477 | 1.68 | 0.1227 |
| APOC2 | 344 | 2.37 | 0.1254 |
| ANXA6 | 309 | 1.97 | 0.1273 |
| ACTBL2 | 345651 | -2.46 | 0.1278 |
| CERS2 | 29956 | -2.60 | 0.1297 |
| RAB5C | 5878 | 1.92 | 0.1325 |
| CORO1A | 11151 | -1.60 | 0.1332 |
| PLXDC2 | 84898 | -1.13 | 0.1336 |
| CSRP1 | 1465 | -2.01 | 0.1382 |
| MLEC | 9761 | -2.15 | 0.1391 |
| SDR42E1 | 93517 | -3.57 | 0.1424 |
| CFHR1 | 3078 | -2.87 | 0.1449 |
| RSU1 | 6251 | -2.69 | 0.1474 |
| HSPA1A | 3303 | 1.08 | 0.1494 |
| HSPA1B | 3304 | 1.08 | 0.1494 |
| TXN | 7295 | 1.50 | 0.1525 |
| ARPC3 | 10094 | -2.32 | 0.1536 |
| GLUD1 | 2746 | -1.45 | 0.1557 |
| TUFM | 7284 | 1.82 | 0.1574 |
| CALML3 | 810 | 1.52 | 0.1589 |
| CALM2 | 805 | 1.59 | 0.1601 |
| CALM1 | 801 | 1.60 | 0.1603 |
| CALM3 | 808 | 1.63 | 0.1608 |
| CALR | 811 | -1.40 | 0.1623 |
| PLP2 | 5355 | 1.62 | 0.1648 |
| GIMAP1 | 170575 | -0.90 | 0.1648 |
| GIMAP1-GIMAP5 | 100527949 | -0.84 | 0.1648 |
| RPL10A | 4736 | 0.91 | 0.1648 |
| COX2 | 4513 | 1.40 | 0.1649 |
| PTPRCAP | 5790 | -0.03 | 0.1650 |
| LBP | 3929 | -1.04 | 0.1650 |
| CDC42 | 998 | 1.41 | 0.1651 |
| GPX3 | 2878 | 1.08 | 0.1651 |
| DLD | 1738 | 1.11 | 0.1652 |
| ICAM2 | 3384 | -1.12 | 0.1652 |
| TMX3 | 54495 | -0.89 | 0.1652 |
| CAP2 | 10486 | -1.17 | 0.1653 |
| NDUFV1 | 4723 | 0.94 | 0.1656 |
| LTN1 | 26046 | 1.41 | 0.1656 |
| PRDX5 | 25824 | 1.45 | 0.1657 |
| TECR | 9524 | 0.98 | 0.1661 |
| LRBA | 987 | 0.61 | 0.1662 |
| CCDC138 | 165055 | -1.37 | 0.1663 |
| PARVA | 55742 | -0.99 | 0.1664 |
| PARVB | 29780 | -2.28 | 0.1674 |
| MGST3 | 4259 | 1.02 | 0.1674 |
| ABCB11 | 8647 | -0.75 | 0.1676 |
| SAA2 | 6289 | 1.25 | 0.1676 |
| PDE5A | 8654 | 0.81 | 0.1684 |
| ERP29 | 10961 | 1.21 | 0.1687 |
| SCAMP2 | 10066 | 1.37 | 0.1688 |
| COPA | 1314 | 0.79 | 0.1691 |
| ANO6 | 196527 | 0.94 | 0.1694 |
| NFATC3 | 4775 | -1.24 | 0.1708 |
| ABHD16A | 7920 | 0.96 | 0.1711 |
| HSP90AB1 | 3326 | -0.93 | 0.1719 |
| CAPN1 | 823 | 0.91 | 0.1720 |
| H2AFV | 94239 | 2.19 | 0.1733 |
| H2AFZ | 3015 | 2.15 | 0.1744 |
| IGFALS | 3483 | 1.06 | 0.1745 |
| GPI | 2821 | 1.37 | 0.1745 |
| BCAP31 | 10134 | -2.15 | 0.1747 |
| RPS14 | 6208 | -2.08 | 0.1747 |
| SLC25A5 | 292 | -1.46 | 0.1817 |
| HRG | 3273 | -1.57 | 0.1890 |
| MSN | 4478 | -1.40 | 0.1911 |
| TUBB1 | 81027 | 0.47 | 0.1916 |
| FLOT2 | 2319 | -1.16 | 0.1935 |
| TMX4 | 56255 | -0.98 | 0.1979 |
| SMIM1 | 388588 | -1.03 | 0.1991 |
| ARHGAP35 | 2909 | 2.26 | 0.2082 |
| PHB | 5245 | 1.47 | 0.2116 |
| ACTG1 | 71 | 2.16 | 0.2136 |
| CYB5B | 80777 | -1.54 | 0.2179 |
| RPL23A | 6147 | -1.97 | 0.2181 |
| EEF1A1 | 1915 | 1.52 | 0.2188 |
| EEF1A2 | 1917 | 1.52 | 0.2188 |
| ACTA1 | 58 | -1.70 | 0.2285 |
| PDHB | 5162 | 1.48 | 0.2297 |
| TREML1 | 340205 | -1.30 | 0.2300 |
| RBP4 | 5950 | 2.21 | 0.2313 |
| HLA-B | 3106 | -0.37 | 0.2326 |
| HSPA9 | 3313 | -1.15 | 0.2341 |
| STXBP2 | 6813 | 1.07 | 0.2375 |
| FCER1G | 2207 | 1.02 | 0.2379 |
| TMX1 | 81542 | 2.48 | 0.2446 |
| RPL4 | 6124 | -2.06 | 0.2456 |
| CFI | 3426 | 1.10 | 0.2471 |
| YWHAG | 7532 | 1.67 | 0.2487 |
| P2RX1 | 5023 | -1.33 | 0.2493 |
| RPS4X | 6191 | -1.38 | 0.2498 |
| CPN2 | 1370 | 1.10 | 0.2504 |
| CS | 1431 | -0.96 | 0.2505 |
| SDPR | 8436 | -0.55 | 0.2513 |
| HMGA1 | 3159 | -1.94 | 0.2515 |
| RAB14 | 51552 | -2.20 | 0.2521 |
| ACTR3 | 10096 | -2.30 | 0.2523 |
| PECAM1 | 5175 | -0.38 | 0.2527 |
| ALB | 213 | 0.22 | 0.2539 |
| TUBB4B | 10383 | -2.35 | 0.2554 |
| MNDA | 4332 | -1.59 | 0.2557 |
| FGA | 2243 | -0.16 | 0.2561 |
| MYO18A | 399687 | -0.51 | 0.2575 |
| ATP4A | 495 | -0.27 | 0.2584 |
| ATP1A2 | 477 | -0.28 | 0.2594 |
| C6 | 729 | -0.78 | 0.2636 |
| WTH3DI | 150786 | 0.84 | 0.2637 |
| RAB6C | 84084 | 0.84 | 0.2637 |
| CHCHD2 | 51142 | 1.69 | 0.2651 |
| ACTR1A | 10121 | -1.41 | 0.2678 |
| C1QBP | 708 | -1.62 | 0.2679 |
| SPCS1 | 28972 | -0.82 | 0.2688 |
| ENO1 | 2023 | -0.79 | 0.2763 |
| EMILIN1 | 11117 | 1.54 | 0.2780 |
| PON1 | 5444 | 0.88 | 0.2792 |
| RAB11B | 9230 | 1.30 | 0.2830 |
| LYN | 4067 | -1.56 | 0.2836 |
| AMBP | 259 | 0.26 | 0.2840 |
| RAB11A | 8766 | 1.34 | 0.2841 |
| PROS1 | 5627 | -1.03 | 0.2875 |
| ORM2 | 5005 | -1.47 | 0.2895 |
| MYLK | 4638 | -0.32 | 0.2898 |
| CKM | 1158 | -0.80 | 0.2902 |
| RDH11 | 51109 | 1.03 | 0.2924 |
| TUBB | 203068 | 1.08 | 0.2942 |
| IQGAP1 | 8826 | 0.30 | 0.2947 |
| SACM1L | 22908 | 1.49 | 0.2970 |
| SAA2-SAA4 | 100528017 | -0.44 | 0.2984 |
| SAA4 | 6291 | -0.44 | 0.2984 |
| AK2 | 204 | -0.80 | 0.2988 |
| SERPING1 | 710 | -1.18 | 0.3001 |
| HSPA6 | 3310 | -0.77 | 0.3022 |
| ATP5D | 513 | -1.85 | 0.3087 |
| RPL26L1 | 51121 | 1.35 | 0.3098 |
| RPL26 | 6154 | 1.35 | 0.3098 |
| MYL12A | 10627 | -1.27 | 0.3152 |
| MYL12B | 103910 | -1.28 | 0.3152 |
| VASP | 7408 | -1.38 | 0.3188 |
| SQRDL | 58472 | 0.39 | 0.3188 |
| A1BG | 1 | -0.42 | 0.3214 |
| FN1 | 2335 | 0.12 | 0.3217 |
| PLG | 5340 | -0.24 | 0.3231 |
| MRVI1 | 10335 | -0.87 | 0.3244 |
| ECM1 | 1893 | -0.96 | 0.3252 |
| APOL1 | 8542 | -0.86 | 0.3256 |
| ACADM | 34 | -0.62 | 0.3265 |
| CLEC3B | 7123 | -1.54 | 0.3324 |
| RAB5B | 5869 | 0.04 | 0.3356 |
| FAU | 2197 | 0.05 | 0.3356 |
| RPL35 | 11224 | 0.12 | 0.3356 |
| MYH7B | 57644 | 0.16 | 0.3356 |
| ARHGAP30 | 257106 | -0.27 | 0.3356 |
| MYH8 | 4626 | 0.17 | 0.3356 |
| INF2 | 64423 | 0.27 | 0.3356 |
| TKT | 7086 | 0.29 | 0.3356 |
| LBR | 3930 | 0.06 | 0.3356 |
| ALOX5AP | 241 | -0.73 | 0.3356 |
| EEF2 | 1938 | -0.48 | 0.3356 |
| TRIM58 | 25893 | -0.42 | 0.3356 |
| PROC | 5624 | -0.40 | 0.3356 |
| SDHB | 6390 | 0.47 | 0.3356 |
| NDUFA9 | 4704 | -0.44 | 0.3356 |
| STT3A | 3703 | -0.42 | 0.3356 |
| TMED7-TICAM2 | 100302736 | 0.39 | 0.3356 |
| TOR4A | 54863 | 0.37 | 0.3356 |
| TCP1 | 6950 | 0.34 | 0.3356 |
| CUL4B | 8450 | 0.32 | 0.3356 |
| RPS2 | 6187 | 0.58 | 0.3356 |
| HERC2 | 8924 | 0.55 | 0.3356 |
| CA2 | 760 | 0.62 | 0.3356 |
| RPS7 | 6201 | -0.63 | 0.3356 |
| SAFB | 6294 | -0.45 | 0.3356 |
| NICN1 | 84276 | -0.75 | 0.3356 |
| PHKB | 5257 | -0.72 | 0.3356 |
| LRRFIP1 | 9208 | -0.38 | 0.3356 |
| DCP2 | 167227 | -0.62 | 0.3356 |
| CFHR3 | 10878 | 0.72 | 0.3356 |
| ITPR1 | 3708 | 0.34 | 0.3356 |
| KRT14 | 3861 | 0.60 | 0.3356 |
| RAP2C | 57826 | 0.56 | 0.3356 |
| TUBB2A | 7280 | 0.66 | 0.3356 |
| C6orf25 | 80739 | 0.71 | 0.3356 |
| CNDP1 | 84735 | 0.47 | 0.3356 |
| NIPSNAP1 | 8508 | 1.07 | 0.3356 |
| HVCN1 | 84329 | -0.56 | 0.3356 |
| NDUFB10 | 4716 | -0.60 | 0.3356 |
| STOML2 | 30968 | 0.44 | 0.3356 |
| LCK | 3932 | -0.43 | 0.3356 |
| HACD4 | 401494 | 0.47 | 0.3356 |
| ATP6 | 4508 | 0.74 | 0.3356 |
| PCCB | 5096 | -0.63 | 0.3356 |
| TESC | 54997 | -0.54 | 0.3356 |
| CISD1 | 55847 | -0.67 | 0.3356 |
| RAB5A | 5868 | 0.04 | 0.3356 |
| RPS11 | 6205 | 0.68 | 0.3356 |
| SNCB | 6620 | -0.60 | 0.3356 |
| TSPO | 706 | 0.78 | 0.3356 |
| MEIKIN | 728637 | -0.61 | 0.3356 |
| SNX25 | 83891 | -0.58 | 0.3356 |
| WASL | 8976 | -0.74 | 0.3356 |
| CD14 | 929 | 0.47 | 0.3356 |
| CNN3 | 1266 | 0.47 | 0.3356 |
| HNRNPA1 | 3178 | -0.41 | 0.3356 |
| RPL29 | 6159 | -0.73 | 0.3356 |
| CIT | 11113 | -0.64 | 0.3356 |
| TAS2R42 | 353164 | -0.97 | 0.3356 |
| COX17 | 10063 | 0.58 | 0.3356 |
| COPE | 11316 | 0.53 | 0.3356 |
| ENO3 | 2027 | 0.51 | 0.3356 |
| FKBP1A | 2280 | 0.74 | 0.3356 |
| GFAP | 2670 | 0.57 | 0.3356 |
| GNAS | 2778 | 0.39 | 0.3356 |
| TUBB2B | 347733 | 0.65 | 0.3356 |
| UBE2NL | 389898 | 0.51 | 0.3356 |
| NDUFB9 | 4715 | 0.64 | 0.3356 |
| TNFRSF11B | 4982 | 0.49 | 0.3356 |
| CYCS | 54205 | 0.68 | 0.3356 |
| UGGT1 | 56886 | 0.35 | 0.3356 |
| RAP2A | 5911 | 0.55 | 0.3356 |
| RAP2B | 5912 | 0.55 | 0.3356 |
| SH3BGRL | 6451 | 0.59 | 0.3356 |
| SUMO2 | 6613 | 0.57 | 0.3356 |
| UBE2N | 7334 | 0.53 | 0.3356 |
| MAP7D3 | 79649 | 0.47 | 0.3356 |
| RNGTT | 8732 | 0.64 | 0.3356 |
| TAPBP | 6892 | 0.59 | 0.3356 |
| RPS19 | 6223 | -0.60 | 0.3356 |
| ARPC4-TTLL3 | 100526693 | 0.57 | 0.3356 |
| TAOK3 | 51347 | 0.47 | 0.3356 |
| HPR | 3250 | 0.82 | 0.3356 |
| ITGB2 | 3689 | -0.46 | 0.3356 |
| RPL9 | 6133 | -0.46 | 0.3356 |
| ELOVL7 | 79993 | -0.44 | 0.3356 |
| ATPIF1 | 93974 | 0.71 | 0.3356 |
| CTSC | 1075 | -0.58 | 0.3356 |
| SEC61B | 10952 | -0.59 | 0.3356 |
| PARP1 | 142 | -0.38 | 0.3356 |
| GABRA4 | 2557 | -0.57 | 0.3356 |
| SAMM50 | 25813 | -0.34 | 0.3356 |
| FETUB | 26998 | 0.44 | 0.3356 |
| GRM5 | 2915 | -0.53 | 0.3356 |
| TUBB8 | 347688 | -0.64 | 0.3356 |
| NDUFS3 | 4722 | -0.38 | 0.3356 |
| ACOX1 | 51 | -0.44 | 0.3356 |
| CLEC1B | 51266 | -0.61 | 0.3356 |
| BSG | 682 | -0.59 | 0.3356 |
| KRT6B | 3854 | 0.63 | 0.3356 |
| RPL3 | 6122 | 0.57 | 0.3356 |
| TMPO | 7112 | 0.35 | 0.3356 |
| HNRNPU | 3192 | 0.19 | 0.3356 |
| TWF2 | 11344 | -0.51 | 0.3356 |
| RPS3A | 6189 | -0.44 | 0.3356 |
| RPS16 | 6217 | -0.65 | 0.3356 |
| UTS2 | 10911 | -0.88 | 0.3356 |
| PRDX3 | 10935 | 0.47 | 0.3356 |
| SLC27A3 | 11000 | 0.37 | 0.3356 |
| DYNLL2 | 140735 | 0.55 | 0.3356 |
| ALOX12 | 239 | 0.40 | 0.3356 |
| ATP2C1 | 27032 | 0.33 | 0.3356 |
| GNAI3 | 2773 | 0.41 | 0.3356 |
| LTF | 4057 | 0.66 | 0.3356 |
| NDUFS4 | 4724 | 0.60 | 0.3356 |
| TMED7 | 51014 | 0.43 | 0.3356 |
| PCOLCE | 5118 | 0.58 | 0.3356 |
| MAP7D1 | 55700 | 0.44 | 0.3356 |
| AZGP1 | 563 | 0.56 | 0.3356 |
| AKR7A2 | 8574 | 0.48 | 0.3356 |
| DYNLL1 | 8655 | 0.55 | 0.3356 |
| BANF1 | 8815 | 0.64 | 0.3356 |
| MAP4K4 | 9448 | 0.51 | 0.3356 |
| N4BP1 | 9683 | 0.82 | 0.3356 |
| MAOA | 4128 | 0.39 | 0.3356 |
| SYTL4 | 94121 | -0.51 | 0.3356 |
| HMGCL | 3155 | 0.42 | 0.3356 |
| SAFB2 | 9667 | -0.45 | 0.3356 |
| H2AFY2 | 55506 | -0.41 | 0.3356 |
| PRG4 | 10216 | 0.41 | 0.3356 |
| CPA3 | 1359 | 0.71 | 0.3356 |
| TOR1AIP1 | 26092 | 0.40 | 0.3356 |
| GNAI1 | 2770 | 0.42 | 0.3356 |
| GNAL | 2774 | 0.43 | 0.3356 |
| EYS | 346007 | 0.44 | 0.3356 |
| KPNA2 | 3838 | 0.69 | 0.3356 |
| SUMO4 | 387082 | 0.55 | 0.3356 |
| RPL7 | 6129 | 0.42 | 0.3356 |
| SUMO3 | 6612 | 0.55 | 0.3356 |
| MAP7 | 9053 | 0.45 | 0.3356 |
| CTTN | 2017 | 0.54 | 0.3356 |
| GNAO1 | 2775 | 0.41 | 0.3356 |
| GNAT1 | 2779 | 0.41 | 0.3356 |
| GNAT2 | 2780 | 0.40 | 0.3356 |
| GNAT3 | 346562 | 0.41 | 0.3356 |
| SNAP29 | 9342 | 0.38 | 0.3356 |
| GAL3ST1 | 9514 | 0.63 | 0.3356 |
| GRPEL1 | 80273 | 0.07 | 0.3356 |
| IARS2 | 55699 | 0.09 | 0.3356 |
| CST3 | 1471 | -0.03 | 0.3356 |
| ERAP1 | 51752 | 0.02 | 0.3356 |
| METTL7A | 25840 | 0.07 | 0.3356 |
| ADIPOQ | 9370 | -0.03 | 0.3356 |
| RPL19 | 6143 | 0.05 | 0.3356 |
| C4A | 720 | -0.92 | 0.3362 |
| HSPA1L | 3305 | 0.71 | 0.3372 |
| HMGB2 | 3148 | -1.50 | 0.3401 |
| C22orf23 | 84645 | -2.04 | 0.3438 |
| C2 | 717 | 1.38 | 0.3444 |
| GSN | 2934 | -0.18 | 0.3449 |
| ATP5A1 | 498 | -0.29 | 0.3465 |
| HIST2H3C | 126961 | -0.89 | 0.3475 |
| HIST2H3A | 333932 | -0.89 | 0.3475 |
| HIST2H3D | 653604 | -0.89 | 0.3475 |
| HIST1H3A | 8350 | -0.89 | 0.3475 |
| HIST1H3D | 8351 | -0.89 | 0.3475 |
| HIST1H3C | 8352 | -0.89 | 0.3475 |
| HIST1H3E | 8353 | -0.89 | 0.3475 |
| HIST1H3I | 8354 | -0.89 | 0.3475 |
| HIST1H3G | 8355 | -0.89 | 0.3475 |
| HIST1H3J | 8356 | -0.89 | 0.3475 |
| HIST1H3H | 8357 | -0.89 | 0.3475 |
| HIST1H3B | 8358 | -0.89 | 0.3475 |
| HIST1H3F | 8968 | -0.89 | 0.3475 |
| RAB7A | 7879 | -1.20 | 0.3486 |
| TGFB1 | 7040 | -0.56 | 0.3503 |
| FBLN1 | 2192 | 1.68 | 0.3504 |
| TAP2 | 6891 | -0.95 | 0.3506 |
| RAP1B | 5908 | -1.08 | 0.3518 |
| C4B_2 | 100293534 | -0.85 | 0.3522 |
| C4B | 721 | -0.85 | 0.3522 |
| EEF1B2 | 1933 | -1.03 | 0.3531 |
| SLC25A6 | 293 | -1.37 | 0.3543 |
| ACTB | 60 | -0.37 | 0.3546 |
| F2 | 2147 | -0.73 | 0.3553 |
| CPT1A | 1374 | -0.85 | 0.3573 |
| ESAM | 90952 | -1.18 | 0.3574 |
| LGALS3BP | 3959 | 0.49 | 0.3575 |
| C1QA | 712 | -1.40 | 0.3586 |
| SEC22B | 9554 | 1.60 | 0.3603 |
| FGB | 2244 | -0.12 | 0.3613 |
| ROCK2 | 9475 | 0.61 | 0.3626 |
| RAB18 | 22931 | 0.41 | 0.3641 |
| CD226 | 10666 | 1.30 | 0.3703 |
| SERPIND1 | 3053 | -0.24 | 0.3707 |
| TUBA1A | 7846 | -0.80 | 0.3722 |
| TUBA1B | 10376 | -0.79 | 0.3723 |
| CALD1 | 800 | 1.23 | 0.3736 |
| YWHAZ | 7534 | -0.26 | 0.3738 |
| SCCPDH | 51097 | -0.44 | 0.3770 |
| FHL1 | 2273 | -0.50 | 0.3804 |
| IFI16 | 3428 | -0.31 | 0.3809 |
| APOA2 | 336 | -0.95 | 0.3816 |
| FHOD1 | 29109 | 0.51 | 0.3837 |
| GP5 | 2814 | -1.19 | 0.3839 |
| SERPINA6 | 866 | -1.79 | 0.3851 |
| MYL1 | 4632 | -0.28 | 0.3872 |
| LRRC59 | 55379 | 1.28 | 0.3897 |
| MAOB | 4129 | 1.23 | 0.3908 |
| TF | 7018 | 0.14 | 0.3928 |
| ACTR3B | 57180 | -1.10 | 0.3929 |
| RPN2 | 6185 | 1.34 | 0.3946 |
| VAPA | 9218 | -1.10 | 0.3952 |
| PPIF | 10105 | -1.74 | 0.3956 |
| CD74 | 972 | -0.99 | 0.3961 |
| HIST1H2BD | 3017 | -0.33 | 0.3970 |
| HIST2H2BF | 440689 | -0.33 | 0.3970 |
| LOC102724334 | 102724334 | -0.33 | 0.3970 |
| H2BFS | 54145 | -0.33 | 0.3970 |
| HIST1H2BG | 8339 | -0.33 | 0.3970 |
| HIST1H2BN | 8341 | -0.33 | 0.3970 |
| HIST1H2BM | 8342 | -0.33 | 0.3970 |
| HIST1H2BF | 8343 | -0.33 | 0.3970 |
| HIST1H2BE | 8344 | -0.33 | 0.3970 |
| HIST1H2BH | 8345 | -0.33 | 0.3970 |
| HIST1H2BI | 8346 | -0.33 | 0.3970 |
| HIST1H2BC | 8347 | -0.33 | 0.3970 |
| HIST1H2BK | 85236 | -0.33 | 0.3970 |
| SPTBN1 | 6711 | -0.48 | 0.3981 |
| ACTN1 | 87 | -0.48 | 0.3989 |
| SDHA | 6389 | 0.89 | 0.4013 |
| LMAN2 | 10960 | 0.90 | 0.4028 |
| SUN2 | 25777 | 0.78 | 0.4114 |
| ATP1A1 | 476 | 0.68 | 0.4121 |
| CAPZA1 | 829 | -0.27 | 0.4126 |
| SAA1 | 6288 | 0.61 | 0.4129 |
| CST7 | 8530 | 2.21 | 0.4129 |
| ETFB | 2109 | -1.37 | 0.4184 |
| HSPA8 | 3312 | -0.61 | 0.4187 |
| HIST2H2AB | 317772 | 1.48 | 0.4187 |
| HYOU1 | 10525 | 0.66 | 0.4198 |
| MECP2 | 4204 | -0.60 | 0.4265 |
| TTR | 7276 | 0.23 | 0.4291 |
| GP1BB | 2812 | -1.40 | 0.4293 |
| C9 | 735 | -1.07 | 0.4304 |
| RPL28 | 6158 | -1.13 | 0.4305 |
| H2AFX | 3014 | -1.36 | 0.4338 |
| HIST1H1D | 3007 | -1.29 | 0.4372 |
| ACTG2 | 72 | 2.34 | 0.4381 |
| ACTA2 | 59 | 2.32 | 0.4381 |
| S100A10 | 6281 | 1.07 | 0.4382 |
| ZMPSTE24 | 10269 | -1.04 | 0.4394 |
| ESYT1 | 23344 | 0.99 | 0.4444 |
| 44445 | 23157 | 0.60 | 0.4444 |
| ABRACL | 58527 | 1.13 | 0.4468 |
| VCL | 7414 | -0.41 | 0.4488 |
| AFM | 173 | -0.60 | 0.4520 |
| KRT10 | 3858 | -1.01 | 0.4524 |
| GSTK1 | 373156 | -0.74 | 0.4552 |
| UQCRB | 7381 | -1.06 | 0.4562 |
| GP9 | 2815 | -0.24 | 0.4586 |
| YWHAB | 7529 | 1.50 | 0.4616 |
| RAB10 | 10890 | -1.35 | 0.4635 |
| USMG5 | 84833 | -1.49 | 0.4649 |
| YWHAQ | 10971 | 0.57 | 0.4650 |
| TPM3 | 7170 | -0.23 | 0.4653 |
| EWSR1 | 2130 | -0.05 | 0.4708 |
| GPX1 | 2876 | -1.03 | 0.4723 |
| TPM1 | 7168 | 0.97 | 0.4741 |
| CD84 | 8832 | 0.75 | 0.4747 |
| NAPA | 8775 | 0.62 | 0.4751 |
| APOL3 | 80833 | 1.31 | 0.4752 |
| THBS1 | 7057 | -0.22 | 0.4753 |
| YWHAE | 7531 | 1.34 | 0.4753 |
| ARL8B | 55207 | 0.63 | 0.4770 |
| MYL6 | 4637 | -0.23 | 0.4777 |
| ACO2 | 50 | -0.80 | 0.4798 |
| PDIA3 | 2923 | -0.19 | 0.4815 |
| HSP90B1 | 7184 | -0.28 | 0.4825 |
| ILK | 3611 | -0.78 | 0.4832 |
| VAMP3 | 9341 | -0.96 | 0.4859 |
| CLU | 1191 | 0.16 | 0.4877 |
| HNRNPH2 | 3188 | 0.51 | 0.4884 |
| MYH4 | 4622 | -0.28 | 0.4891 |
| CYBA | 1535 | 0.84 | 0.4901 |
| PDLIM1 | 9124 | -1.40 | 0.4906 |
| DLST | 1743 | 0.84 | 0.4908 |
| HNRNPH1 | 3187 | 0.52 | 0.4909 |
| EHD3 | 30845 | 0.74 | 0.4920 |
| F13A1 | 2162 | 0.20 | 0.4923 |
| SLC25A3 | 5250 | -1.39 | 0.4964 |
| MYL3 | 4634 | -0.21 | 0.4989 |
| LDHA | 3939 | -0.91 | 0.4998 |
| GLS | 2744 | -1.13 | 0.4998 |
| F12 | 2161 | 0.51 | 0.5019 |
| ACTC1 | 70 | -1.49 | 0.5050 |
| PPBP | 5473 | -0.30 | 0.5064 |
| KIT | 3815 | -0.77 | 0.5095 |
| KNG1 | 3827 | -0.95 | 0.5164 |
| CMTM5 | 116173 | -0.98 | 0.5166 |
| STAU1 | 6780 | 0.59 | 0.5178 |
| CP | 1356 | 0.22 | 0.5179 |
| TOMM22 | 56993 | 0.66 | 0.5180 |
| STAU2 | 27067 | 0.60 | 0.5184 |
| RAB27B | 5874 | 0.59 | 0.5210 |
| STOML3 | 161003 | -1.05 | 0.5216 |
| PNP | 4860 | -0.57 | 0.5227 |
| GC | 2638 | 0.77 | 0.5257 |
| IMMT | 10989 | 0.64 | 0.5276 |
| EZR | 7430 | -0.55 | 0.5278 |
| TPM4 | 7171 | -0.23 | 0.5311 |
| NAP1L1 | 4673 | -0.92 | 0.5338 |
| GANAB | 23193 | -0.96 | 0.5345 |
| RDX | 5962 | -0.57 | 0.5354 |
| IQGAP2 | 10788 | 0.48 | 0.5401 |
| TMEM40 | 55287 | -1.04 | 0.5409 |
| ITGB3 | 3690 | -0.28 | 0.5426 |
| CLTC | 1213 | -0.57 | 0.5458 |
| HSPD1 | 3329 | -0.20 | 0.5464 |
| ECH1 | 1891 | -1.10 | 0.5465 |
| OGDHL | 55753 | -0.37 | 0.5499 |
| HSPA5 | 3309 | -0.17 | 0.5504 |
| IDH2 | 3418 | -0.90 | 0.5506 |
| MYL10 | 93408 | -1.13 | 0.5511 |
| HABP2 | 3026 | -0.80 | 0.5516 |
| C1R | 715 | -0.98 | 0.5518 |
| COX5A | 9377 | 0.79 | 0.5533 |
| TST | 7263 | -1.12 | 0.5587 |
| APOB | 338 | -0.16 | 0.5589 |
| HBD | 3045 | -0.86 | 0.5605 |
| ME2 | 4200 | -0.66 | 0.5619 |
| EIF5A | 1984 | -0.68 | 0.5621 |
| NDUFS1 | 4719 | 0.44 | 0.5647 |
| EBF2 | 64641 | 0.40 | 0.5650 |
| ERP44 | 23071 | -0.50 | 0.5658 |
| NDUFV2 | 4729 | 0.56 | 0.5700 |
| UQCRC1 | 7384 | -0.56 | 0.5715 |
| TMED9 | 54732 | 0.66 | 0.5720 |
| SERPINA3 | 12 | 0.52 | 0.5772 |
| CHMP4A | 29082 | 0.81 | 0.5772 |
| HSPE1 | 3336 | -1.07 | 0.5794 |
| APOC1 | 341 | -0.10 | 0.5801 |
| CAPZB | 832 | -0.77 | 0.5844 |
| FGG | 2266 | -0.07 | 0.5845 |
| ACADVL | 37 | 0.38 | 0.5851 |
| HSD17B10 | 3028 | 0.60 | 0.5854 |
| SERPINC1 | 462 | 0.22 | 0.5857 |
| SLC25A11 | 8402 | -0.42 | 0.5867 |
| ARPC4 | 10093 | -1.01 | 0.5868 |
| GAPDH | 2597 | -0.57 | 0.5869 |
| TUBB3 | 10381 | -1.40 | 0.5949 |
| GP6 | 51206 | 0.49 | 0.5952 |
| HSPE1-MOB4 | 100529241 | -0.55 | 0.5953 |
| RER1 | 11079 | -0.78 | 0.5959 |
| LMNA | 4000 | -0.41 | 0.5976 |
| MYH9 | 4627 | -0.18 | 0.5996 |
| VTN | 7448 | -0.12 | 0.6003 |
| MYH6 | 4624 | -0.24 | 0.6009 |
| RAC2 | 5880 | -0.62 | 0.6014 |
| RAC3 | 5881 | -0.62 | 0.6014 |
| MYH7 | 4625 | -0.24 | 0.6019 |
| RAC1 | 5879 | -0.60 | 0.6020 |
| ACAT1 | 38 | -0.65 | 0.6022 |
| 44446 | 989 | 0.48 | 0.6037 |
| APCS | 325 | 0.53 | 0.6038 |
| DPM3 | 54344 | 0.60 | 0.6046 |
| NDUFB11 | 54539 | -0.70 | 0.6054 |
| DMD | 1756 | -0.73 | 0.6056 |
| HSD17B12 | 51144 | -0.72 | 0.6067 |
| GNB3 | 2784 | 0.48 | 0.6070 |
| GPD2 | 2820 | -0.74 | 0.6072 |
| SERPINF2 | 5345 | -0.15 | 0.6083 |
| PARK7 | 11315 | -0.55 | 0.6093 |
| DCTN1 | 1639 | 0.24 | 0.6105 |
| WDR1 | 9948 | -0.53 | 0.6106 |
| ATL3 | 25923 | 0.55 | 0.6114 |
| TUBA8 | 51807 | 0.77 | 0.6122 |
| CFB | 629 | 0.11 | 0.6126 |
| TPI1 | 7167 | -0.84 | 0.6143 |
| IER3IP1 | 51124 | 0.59 | 0.6160 |
| PRKAR1A | 5573 | -0.43 | 0.6162 |
| HMGB1 | 3146 | -0.98 | 0.6168 |
| ATP5F1 | 515 | 0.63 | 0.6169 |
| MDH2 | 4191 | -0.48 | 0.6178 |
| F11R | 50848 | 0.70 | 0.6178 |
| SPARC | 6678 | 0.72 | 0.6185 |
| C1RL | 51279 | -0.49 | 0.6187 |
| SPERT | 220082 | -0.58 | 0.6207 |
| ZYX | 7791 | -0.47 | 0.6222 |
| TMOD3 | 29766 | -0.68 | 0.6242 |
| DBNL | 28988 | 0.36 | 0.6268 |
| STX11 | 8676 | 0.68 | 0.6271 |
| PGK1 | 5230 | 0.64 | 0.6273 |
| S100A4 | 6275 | 0.82 | 0.6301 |
| PF4 | 5196 | -0.20 | 0.6369 |
| PF4V1 | 5197 | -0.20 | 0.6369 |
| CMTM6 | 54918 | 0.62 | 0.6386 |
| PRKCB | 5579 | 0.51 | 0.6405 |
| LPA | 4018 | -0.57 | 0.6406 |
| C7 | 730 | -0.54 | 0.6419 |
| AK3 | 50808 | -0.55 | 0.6433 |
| TRIM33 | 51592 | -0.59 | 0.6437 |
| PGRMC2 | 10424 | -1.00 | 0.6437 |
| AGT | 183 | -0.12 | 0.6440 |
| SLMAP | 7871 | 0.33 | 0.6441 |
| AHNAK | 79026 | -0.20 | 0.6448 |
| FLOT1 | 10211 | 0.38 | 0.6477 |
| ITGA2B | 3674 | 0.17 | 0.6487 |
| IGLL5 | 100423062 | 0.56 | 0.6487 |
| JCHAIN | 3512 | 0.59 | 0.6523 |
| EHD1 | 10938 | 0.32 | 0.6529 |
| HNRNPK | 3190 | -0.35 | 0.6531 |
| ACTN4 | 81 | -0.69 | 0.6541 |
| LETM1 | 3954 | -0.28 | 0.6558 |
| GP1BA | 2811 | -0.16 | 0.6569 |
| APOE | 348 | -0.08 | 0.6580 |
| COX4I1 | 1327 | -0.62 | 0.6599 |
| SERPINF1 | 5176 | -0.58 | 0.6599 |
| NEXN | 91624 | 0.54 | 0.6613 |
| MYH3 | 4621 | -0.17 | 0.6654 |
| KRT16 | 3868 | 0.36 | 0.6656 |
| KRT9 | 3857 | 0.49 | 0.6660 |
| C8G | 733 | -0.47 | 0.6669 |
| SNCA | 6622 | 0.40 | 0.6729 |
| VPS13D | 55187 | 1.11 | 0.6745 |
| PFN1 | 5216 | -0.10 | 0.6749 |
| LMNB1 | 4001 | -0.40 | 0.6758 |
| C1S | 716 | -0.10 | 0.6789 |
| APOC3 | 345 | 0.10 | 0.6798 |
| ACAA2 | 10449 | 0.43 | 0.6806 |
| TMSB10 | 9168 | -0.82 | 0.6821 |
| HLA-C | 3107 | -0.85 | 0.6825 |
| HNRNPM | 4670 | 0.29 | 0.6833 |
| TMED2 | 10959 | 0.52 | 0.6853 |
| SFXN1 | 94081 | 0.38 | 0.6855 |
| ATP1A3 | 478 | 0.30 | 0.6865 |
| VAMP8 | 8673 | 0.65 | 0.6884 |
| TUBA4A | 7277 | -0.10 | 0.6911 |
| HK1 | 3098 | 0.27 | 0.6927 |
| CPN1 | 1369 | 0.35 | 0.6971 |
| HSP90AA1 | 3320 | -0.63 | 0.7024 |
| CAP1 | 10487 | 0.59 | 0.7043 |
| PCBP1 | 5093 | -0.51 | 0.7064 |
| ATP5J2 | 9551 | 0.55 | 0.7065 |
| GNAI2 | 2771 | -0.68 | 0.7080 |
| YBX1 | 4904 | 0.54 | 0.7091 |
| HNRNPA3 | 220988 | 0.35 | 0.7112 |
| RPS18 | 6222 | -0.51 | 0.7182 |
| SPCS2 | 9789 | 0.46 | 0.7186 |
| NDUFA5 | 4698 | -0.58 | 0.7197 |
| SLC4A1 | 6521 | 0.25 | 0.7213 |
| H3F3A | 3020 | -0.77 | 0.7215 |
| H3F3B | 3021 | -0.77 | 0.7215 |
| MYH1 | 4619 | 0.25 | 0.7228 |
| LMNB2 | 84823 | -0.36 | 0.7244 |
| LTBP1 | 4052 | 0.17 | 0.7245 |
| ATP5J2-PTCD1 | 100526740 | 0.32 | 0.7303 |
| B2M | 567 | 0.68 | 0.7352 |
| ARF1 | 375 | 0.62 | 0.7376 |
| ARF3 | 377 | 0.63 | 0.7382 |
| C3 | 718 | 0.05 | 0.7391 |
| CORO1C | 23603 | -0.49 | 0.7423 |
| LOC102724023 | 102724023 | -0.45 | 0.7432 |
| C21orf33 | 8209 | -0.45 | 0.7435 |
| ITIH3 | 3699 | -0.53 | 0.7455 |
| TPM2 | 7169 | 0.68 | 0.7467 |
| C8B | 732 | 0.39 | 0.7481 |
| FLNA | 2316 | 0.07 | 0.7492 |
| LCP1 | 3936 | -0.42 | 0.7503 |
| DBN1 | 1627 | 0.58 | 0.7572 |
| C8A | 731 | -0.46 | 0.7579 |
| ARPC1B | 10095 | -0.50 | 0.7591 |
| C1QC | 714 | 0.62 | 0.7601 |
| ATP5B | 506 | -0.13 | 0.7730 |
| HP | 3240 | 0.05 | 0.7748 |
| CHCHD3 | 54927 | 0.43 | 0.7758 |
| PDIA4 | 9601 | 0.31 | 0.7766 |
| MYH2 | 4620 | -0.21 | 0.7779 |
| MAPRE2 | 10982 | 0.42 | 0.7819 |
| ACTR2 | 10097 | -0.52 | 0.7831 |
| PGAM5 | 192111 | -0.23 | 0.7847 |
| DHRS7 | 51635 | 0.37 | 0.7860 |
| ATP5L | 10632 | -0.62 | 0.7861 |
| CYB5R3 | 1727 | -0.27 | 0.7908 |
| CYFIP1 | 23191 | -0.10 | 0.7912 |
| H2AFY | 9555 | 0.45 | 0.7929 |
| SERPINA7 | 6906 | -0.39 | 0.7934 |
| ORMDL3 | 94103 | 0.38 | 0.7939 |
| PDHA1 | 5160 | -0.32 | 0.7953 |
| RTN4 | 57142 | 0.42 | 0.7971 |
| KLKB1 | 3818 | -0.32 | 0.7995 |
| PPA2 | 27068 | 0.34 | 0.8028 |
| TAP1 | 6890 | -0.26 | 0.8058 |
| RPN1 | 6184 | 0.41 | 0.8078 |
| TGOLN2 | 10618 | -0.26 | 0.8112 |
| RAB27A | 5873 | 0.18 | 0.8125 |
| CCDC168 | 643677 | -0.15 | 0.8135 |
| ECE1 | 1889 | 0.16 | 0.8148 |
| TMEM109 | 79073 | -0.47 | 0.8175 |
| HIST1H1B | 3009 | -0.29 | 0.8192 |
| SEC23A | 10484 | -0.21 | 0.8202 |
| TLN1 | 7094 | -0.06 | 0.8237 |
| PPIA | 5478 | 0.42 | 0.8258 |
| HIST1H1E | 3008 | -0.49 | 0.8273 |
| LGALSL | 29094 | 0.20 | 0.8298 |
| ALDOA | 226 | 0.34 | 0.8337 |
| CECR5 | 27440 | -0.23 | 0.8355 |
| ADAM10 | 102 | 0.36 | 0.8359 |
| APP | 351 | -0.25 | 0.8362 |
| MYH14 | 79784 | 0.08 | 0.8364 |
| YWHAH | 7533 | 0.25 | 0.8376 |
| ITGA6 | 3655 | 0.11 | 0.8377 |
| ORMDL2 | 29095 | 0.16 | 0.8417 |
| CYFIP2 | 26999 | -0.07 | 0.8426 |
| ORMDL1 | 94101 | 0.16 | 0.8456 |
| HIST1H2BL | 8340 | -0.29 | 0.8457 |
| MTDH | 92140 | 0.14 | 0.8457 |
| KRT6C | 286887 | 0.15 | 0.8492 |
| KRT6A | 3853 | 0.15 | 0.8492 |
| CDS2 | 8760 | 0.31 | 0.8502 |
| HBB | 3043 | -0.10 | 0.8503 |
| SAMHD1 | 25939 | -0.14 | 0.8573 |
| CNN2 | 1265 | -0.15 | 0.8575 |
| ATP2A2 | 488 | -0.06 | 0.8585 |
| HIST4H4 | 121504 | -0.10 | 0.8596 |
| HIST2H4B | 554313 | -0.10 | 0.8596 |
| HIST1H4I | 8294 | -0.10 | 0.8596 |
| HIST1H4A | 8359 | -0.10 | 0.8596 |
| HIST1H4D | 8360 | -0.10 | 0.8596 |
| HIST1H4F | 8361 | -0.10 | 0.8596 |
| HIST1H4K | 8362 | -0.10 | 0.8596 |
| HIST1H4J | 8363 | -0.10 | 0.8596 |
| HIST1H4C | 8364 | -0.10 | 0.8596 |
| HIST1H4H | 8365 | -0.10 | 0.8596 |
| HIST1H4B | 8366 | -0.10 | 0.8596 |
| HIST1H4E | 8367 | -0.10 | 0.8596 |
| HIST1H4L | 8368 | -0.10 | 0.8596 |
| HIST2H4A | 8370 | -0.10 | 0.8596 |
| KRT2 | 3849 | -0.17 | 0.8598 |
| LIMS3L | 100288695 | 0.18 | 0.8671 |
| TMED10 | 10972 | 0.23 | 0.8678 |
| ITIH4 | 3700 | -0.04 | 0.8682 |
| FERMT3 | 83706 | -0.28 | 0.8687 |
| TUBA4B | 80086 | -0.04 | 0.8689 |
| CFL1 | 1072 | 0.05 | 0.8699 |
| ITIH1 | 3697 | -0.04 | 0.8710 |
| OPA1 | 4976 | -0.13 | 0.8730 |
| VAMP2 | 6844 | -0.18 | 0.8732 |
| SNAP23 | 8773 | -0.23 | 0.8732 |
| HLA-A | 3105 | -0.27 | 0.8733 |
| STX7 | 8417 | 0.14 | 0.8738 |
| APOOL | 139322 | 0.14 | 0.8765 |
| TUBA1C | 84790 | 0.21 | 0.8767 |
| ATP5O | 539 | -0.28 | 0.8768 |
| PZP | 5858 | 0.16 | 0.8795 |
| RPL17-C18orf32 | 100526842 | 0.13 | 0.8807 |
| S100A6 | 6277 | 0.15 | 0.8812 |
| ITGB1 | 3688 | 0.15 | 0.8833 |
| PHB2 | 11331 | 0.25 | 0.8866 |
| RPL17 | 6139 | 0.13 | 0.8870 |
| DCD | 117159 | -0.20 | 0.8887 |
| ARPC2 | 10109 | 0.26 | 0.8896 |
| FH | 2271 | 0.17 | 0.8907 |
| SH3BGRL3 | 83442 | -0.29 | 0.8909 |
| NNT | 23530 | -0.21 | 0.8919 |
| DDOST | 1650 | 0.17 | 0.8953 |
| SELP | 6403 | -0.23 | 0.8963 |
| CFH | 3075 | -0.02 | 0.9014 |
| ACTRT1 | 139741 | -0.15 | 0.9052 |
| A2M | 2 | 0.03 | 0.9055 |
| NID1 | 4811 | 0.15 | 0.9059 |
| SFXN3 | 81855 | 0.09 | 0.9066 |
| CD9 | 928 | 0.19 | 0.9066 |
| APOA1 | 335 | 0.04 | 0.9068 |
| GSTO1 | 9446 | -0.16 | 0.9102 |
| CYB5A | 1528 | -0.10 | 0.9103 |
| M6PR | 4074 | -0.18 | 0.9105 |
| TBXAS1 | 6916 | -0.12 | 0.9106 |
| GNB1 | 2782 | -0.09 | 0.9110 |
| ARHGDIA | 396 | 0.10 | 0.9115 |
| UQCR10 | 29796 | -0.13 | 0.9128 |
| GNA13 | 10672 | -0.14 | 0.9155 |
| GNA12 | 2768 | -0.14 | 0.9160 |
| MMRN1 | 22915 | -0.03 | 0.9164 |
| NRGN | 4900 | 0.15 | 0.9180 |
| PRKCSH | 5589 | 0.18 | 0.9182 |
| PON3 | 5446 | 0.09 | 0.9202 |
| CANX | 821 | 0.05 | 0.9215 |
| PCBP4 | 57060 | 0.07 | 0.9216 |
| SEPP1 | 6414 | -0.08 | 0.9236 |
| HBA1 | 3039 | -0.05 | 0.9274 |
| HBA2 | 3040 | -0.05 | 0.9274 |
| ENDOD1 | 23052 | 0.08 | 0.9297 |
| SLC2A3 | 6515 | 0.05 | 0.9312 |
| STOM | 2040 | 0.04 | 0.9312 |
| KRT1 | 3848 | 0.10 | 0.9319 |
| HMGN1 | 3150 | -0.08 | 0.9324 |
| VWF | 7450 | 0.05 | 0.9396 |
| LRG1 | 116844 | -0.10 | 0.9406 |
| UQCRC2 | 7385 | -0.09 | 0.9412 |
| H1FX | 8971 | -0.05 | 0.9434 |
| GRB2 | 2885 | 0.05 | 0.9435 |
| HIST1H2AA | 221613 | -0.14 | 0.9440 |
| HPX | 3263 | -0.03 | 0.9444 |
| VCP | 7415 | -0.10 | 0.9447 |
| PTGS1 | 5742 | 0.11 | 0.9450 |
| HIST1H2AE | 3012 | -0.14 | 0.9451 |
| HIST1H2AD | 3013 | -0.14 | 0.9451 |
| H2AFJ | 55766 | -0.14 | 0.9451 |
| HIST2H2AA4 | 723790 | -0.14 | 0.9451 |
| HIST1H2AI | 8329 | -0.14 | 0.9451 |
| HIST1H2AK | 8330 | -0.14 | 0.9451 |
| HIST1H2AJ | 8331 | -0.14 | 0.9451 |
| HIST1H2AL | 8332 | -0.14 | 0.9451 |
| HIST1H2AC | 8334 | -0.14 | 0.9451 |
| HIST1H2AB | 8335 | -0.14 | 0.9451 |
| HIST1H2AM | 8336 | -0.14 | 0.9451 |
| HIST2H2AA3 | 8337 | -0.14 | 0.9451 |
| HIST2H2AC | 8338 | -0.14 | 0.9451 |
| HIST1H2AH | 85235 | -0.14 | 0.9451 |
| HIST1H2AG | 8969 | -0.14 | 0.9451 |
| HIST3H2A | 92815 | -0.14 | 0.9451 |
| C4BPA | 722 | 0.12 | 0.9457 |
| CLIC1 | 1192 | -0.11 | 0.9463 |
| RPS6 | 6194 | -0.07 | 0.9466 |
| 110599563 | 110599563 | -0.06 | 0.9467 |
| ARPC5 | 10092 | 0.09 | 0.9478 |
| ALDOC | 230 | 0.06 | 0.9478 |
| LY6G6F | 259215 | -0.06 | 0.9480 |
| HIST2H3PS2 | 440686 | -0.10 | 0.9505 |
| UQCRFS1 | 7386 | 0.06 | 0.9511 |
| DLAT | 1737 | 0.04 | 0.9528 |
| ARL8A | 127829 | 0.04 | 0.9531 |
| PTPRJ | 5795 | 0.10 | 0.9532 |
| TMED5 | 50999 | 0.05 | 0.9541 |
| TMSB4X | 7114 | 0.01 | 0.9560 |
| ATP2A3 | 489 | 0.02 | 0.9561 |
| CD99 | 4267 | -0.05 | 0.9567 |
| SERPINA1 | 5265 | 0.03 | 0.9567 |
| DECR1 | 1666 | -0.08 | 0.9569 |
| ITIH2 | 3698 | 0.01 | 0.9574 |
| SERPINB1 | 1992 | -0.03 | 0.9580 |
| PRDX1 | 5052 | 0.05 | 0.9598 |
| SFN | 2810 | -0.10 | 0.9604 |
| RAB2A | 5862 | -0.04 | 0.9604 |
| PLEC | 5339 | -0.02 | 0.9612 |
| TAGLN2 | 8407 | -0.06 | 0.9618 |
| C5 | 727 | 0.04 | 0.9635 |
| APOC4 | 346 | 0.05 | 0.9638 |
| MYL5 | 4636 | -0.06 | 0.9644 |
| HNRNPA2B1 | 3181 | -0.06 | 0.9648 |
| RPS13 | 6207 | 0.05 | 0.9656 |
| CAPZA2 | 830 | 0.09 | 0.9661 |
| SH3D19 | 152503 | -0.03 | 0.9665 |
| BIN2 | 51411 | 0.07 | 0.9673 |
| APMAP | 57136 | 0.04 | 0.9688 |
| CD44 | 960 | 0.04 | 0.9693 |
| HIST1H2BB | 3018 | -0.06 | 0.9708 |
| HIST1H2BO | 8348 | -0.06 | 0.9708 |
| HIST2H2BE | 8349 | -0.06 | 0.9708 |
| HIST1H2BJ | 8970 | -0.06 | 0.9708 |
| SSR4 | 6748 | 0.04 | 0.9725 |
| C1QB | 713 | -0.08 | 0.9735 |
| STIM1 | 6786 | -0.02 | 0.9741 |
| ARL6IP5 | 10550 | -0.04 | 0.9745 |
| GSTP1 | 2950 | 0.04 | 0.9758 |
| KIF11 | 3832 | 0.03 | 0.9760 |
| LOC110384692 | 110384692 | -0.06 | 0.9760 |
| P4HB | 5034 | 0.03 | 0.9795 |
| AIFM1 | 9131 | -0.01 | 0.9807 |
| ATP5J | 522 | -0.03 | 0.9815 |
| SFPQ | 6421 | 0.02 | 0.9824 |
| AHSG | 197 | 0.01 | 0.9828 |
| PCBP3 | 54039 | 0.02 | 0.9832 |
| PCBP2 | 5094 | 0.02 | 0.9838 |
| FLNB | 2317 | -0.02 | 0.9847 |
| H3F3C | 440093 | 0.03 | 0.9857 |
| HADHA | 3030 | 0.02 | 0.9862 |
| OGDH | 4967 | -0.01 | 0.9862 |
| GNAQ | 2776 | 0.03 | 0.9864 |
| CA1 | 759 | -0.02 | 0.9892 |
| RAB6B | 51560 | -0.01 | 0.9921 |
| MTPN | 136319 | 0.01 | 0.9924 |
| PTPRC | 5788 | -0.01 | 0.9930 |
| IDH3A | 3419 | -0.01 | 0.9939 |
| RPL18 | 6141 | -0.01 | 0.9944 |
| TMED4 | 222068 | -0.01 | 0.9949 |
| ORM1 | 5004 | 0.00 | 0.9949 |
| UBC | 7316 | -0.01 | 0.9956 |
| UBB | 7314 | -0.01 | 0.9963 |
| UBA52 | 7311 | -0.01 | 0.9966 |
| RPS27A | 6233 | -0.01 | 0.9967 |
| RPS3 | 6188 | 0.01 | 0.9970 |
| HIST3H3 | 8290 | 0.00 | 0.9975 |
| HIST3H2BB | 128312 | 0.00 | 0.9985 |
