# Supplementary material for: Dawn-to-dusk dry fasting induces anti-atherosclerotic, anti-inflammatory, and anti-tumorigenic proteome in peripheral blood mononuclear cells in subjects with metabolic syndrome
Source: Metabol Open. 2022 Nov 1;16:100214. doi: 10.1016/j.metop.2022.100214 (PMC9731888; doi:10.1016/j.metop.2022.100214)
Supplement: Supplementary Table S4 [file mmc4.docx]

| **Supplementary Table S4. Significant Correlations between Fold Changes in PBMC Gene Protein Products and Circulating Components of Metabolic Syndrome, Lipid Panel, Hepatic Panel, and Adiposity, Oxidative Stress and Inflammation Biomarkers at the End of 4-Week Dawn-to-Dusk Dry Fasting Compared with the GP Levels Before 4-Week Dawn-to-Dusk Dry Fasting** | | | | | | | | | | | | | | | |
| --- | --- | --- | --- | --- | --- | --- | --- | --- | --- | --- | --- | --- | --- | --- | --- |
|  | **WaistC (inch)** | **MAP (mmHg)** | **Insulin (µU/ml)** | **TG (mg/dl)** | **TC (mg/dl)** | **ALT (U/L)** | **AST (U/L)** | **GGT (U/L)** | **TBil (mg/dl)** | **ALB (g/dl)** | **TP (g/dl)** | **CRP (mg/L)** | **Leptin (pg/ml)** | **IL-1β (pg/ml)** | **BDNF (ng/ml)** |
| ***Pearson Correlation Coefficient*** | | | | | | | | | | | | | | | |
| ***P Value*** | | | | | | | | | | | | | | | |
| **F10** |  |  |  |  |  |  |  |  |  |  |  |  |  | -0.63 |  |
|  |  |  |  |  |  |  |  |  |  |  |  |  |  | 0.016 |  |
|  | | | | | | | | | | | | | | | |
| **GLUD2** |  |  | -0.53 |  |  |  |  |  |  |  |  |  |  |  |  |
|  |  |  | 0.049 |  |  |  |  |  |  |  |  |  |  |  |  |
|  | | | | | | | | | | | | | | | |
| **TUBB4B** |  |  |  |  |  |  |  |  |  | 0.57 | 0.55 |  |  |  |  |
|  |  |  |  |  |  |  |  |  |  | 0.035 | 0.042 |  |  |  |  |
|  | | | | | | | | | | | | | | | |
| **CFHR2** | -0.60 |  |  |  |  |  |  |  |  |  |  |  |  |  |  |
|  | 0.025 |  |  |  |  |  |  |  |  |  |  |  |  |  |  |
|  | | | | | | | | | | | | | | | |
| **APOL1** |  |  | -0.54 |  |  |  |  | 0.58 |  |  |  |  |  |  | 0.77 |
|  |  |  | 0.046 |  |  |  |  | 0.029 |  |  |  |  |  |  | 0.001 |
|  | | | | | | | | | | | | | | | |
| **HSPA8** |  |  | -0.55 |  |  | 0.66 | 0.57 | 0.65 |  |  |  |  |  |  | 0.68 |
|  |  |  | 0.041 |  |  | 0.010 | 0.032 | 0.012 |  |  |  |  |  |  | 0.007 |
|  | | | | | | | | | | | | | | | |
| **MYL9** |  |  |  |  |  | 0.62 | 0.64 |  |  |  |  |  |  |  |  |
|  |  |  |  |  |  | 0.018 | 0.014 |  |  |  |  |  |  |  |  |
|  | | | | | | | | | | | | | | | |
| **FLNC** |  | -0.55  0.041 |  |  | 0.56  0.037 |  |  |  |  |  |  |  |  |  |  |
|  |  |  |  |  |  |  |  |  |  |  |  |  |  |  |  |
|  | | | | | | | | | | | | | | | |
| **RAB1A** |  |  |  | 0.76 |  |  |  |  | 0.82 | 0.58 | 0.81 |  |  |  |  |
|  |  |  |  | 0.002 |  |  |  |  | 0.0003 | 0.030 | 0.0004 |  |  |  |  |
|  | | | | | | | | | | | | | | | |
| **APOH** |  |  |  |  |  |  |  |  |  |  |  | 0.82 | 0.70 |  |  |
|  |  |  |  |  |  |  |  |  |  |  |  | 0.0003 | 0.005 |  |  |
| WaistC=Waist circumference; MAP=Mean arterial pressure; TG=Triglyceride; TC=Total cholesterol; ALT=Alanine aminotransferase; AST=Aspartate aminotransferase; GGT= Gamma-glutamyl transferase; TBil=Total bilirubin; ALB=Albumin; TP=Total protein; CRP=C-Reactive Protein; IL-1β=Interleukin-1 beta; BDNF=Brain-derived neurotrophic factor | | | | | | | | | | | | | | | |
