# Supplementary material for: Dawn-to-dusk dry fasting induces anti-atherosclerotic, anti-inflammatory, and anti-tumorigenic proteome in peripheral blood mononuclear cells in subjects with metabolic syndrome
Source: Metabol Open. 2022 Nov 1;16:100214. doi: 10.1016/j.metop.2022.100214 (PMC9731888; doi:10.1016/j.metop.2022.100214)
Supplement: Supplementary Table S5 [file mmc5.docx]

| **Supplementary Table S5. Significant Correlations between Fold Changes in PBMC Gene Protein Products and Circulating Components of Metabolic Syndrome, Lipid Panel, Hepatic Panel, and Adiposity, Oxidative Stress and Inflammation Biomarkers One Week after 4-Week Dawn-to-Dusk Dry Fasting Compared with the GP Levels Before 4-Week Dawn-to-Dusk Dry Fasting** | | | | | | | | | | | | | | |
| --- | --- | --- | --- | --- | --- | --- | --- | --- | --- | --- | --- | --- | --- | --- |
|  | **Weight (kg)** | **BMI (kg/m^2^)** | **SBP (mmHg)** | **GLUC (mg/dl)** | **TG (mg/dl)** | **GGT (U/L)** | **ALB (g/dl)** | **TP (g/dl)** | **Leptin (pg/ml)** | **Adipo (µg/ml)** | **CRP (mg/L)** | **IL-6 (pg/ml)** | **HCY (µmol/l)** | **BDNF (ng/ml)** |
| ***Pearson Correlation Coefficient*** | | | | | | | | | | | | | | |
| ***P Value*** | | | | | | | | | | | | | | |
| **H2AFZ** |  |  |  |  |  |  |  |  |  |  |  |  |  | 0.61 |
|  |  |  |  |  |  |  |  |  |  |  |  |  |  | 0.022 |
|  | | | | | | | | | | | | | | |
| **H2AFV** |  |  |  |  |  |  |  |  |  |  |  |  |  | 0.61 |
|  |  |  |  |  |  |  |  |  |  |  |  |  |  | 0.022 |
|  | | | | | | | | | | | | | | |
| **SOD2** |  |  | 0.56 |  |  |  |  |  | -0.55 |  |  |  |  |  |
|  |  |  | 0.037 |  |  |  |  |  | 0.042 |  |  |  |  |  |
|  | | | | | | | | | | | | | | |
| **CP** | 0.65 | 0.65 |  |  |  | 0.55 |  |  |  |  |  |  |  | 0.70 |
|  | 0.011 | 0.011 |  |  |  | 0.040 |  |  |  |  |  |  |  | 0.006 |
|  | | | | | | | | | | | | | | |
| **IGLL5** | 0.64 | 0.64 |  |  |  |  |  |  |  |  |  |  |  | 0.76 |
|  | 0.013 | 0.013 |  |  |  |  |  |  |  |  |  |  |  | 0.002 |
|  | | | | | | | | | | | | | | |
| **KRT77** |  |  |  | 0.73 |  |  |  |  |  |  | 0.89 | 0.64 |  |  |
|  |  |  |  | 0.003 |  |  |  |  |  |  | <0.0001 | 0.013 |  |  |
|  | | | | | | | | | | | | | | |
| **ATP1A3** |  |  |  |  | 0.54 |  |  |  | -0.59 | 0.60 |  |  |  |  |
|  |  |  |  |  | 0.047 |  |  |  | 0.027 | 0.022 |  |  |  |  |
|  | | | | | | | | | | | | | | |
| **APOB** |  |  |  |  |  |  |  |  | -0.56 |  |  |  |  |  |
|  |  |  |  |  |  |  |  |  | 0.039 |  |  |  |  |  |
|  | | | | | | | | | | | | | | |
| **PPIF** |  |  |  |  |  |  |  |  |  |  |  |  | -0.54 |  |
|  |  |  |  |  |  |  |  |  |  |  |  |  | 0.048 |  |
|  | | | | | | | | | | | | | | |
| **FLOT2** |  |  |  |  | -0.71 |  |  |  |  |  |  |  |  |  |
|  |  |  |  |  | 0.004 |  |  |  |  |  |  |  |  |  |
|  | | | | | | | | | | | | | | |
| **ABCB11** |  |  |  |  |  |  | -0.58 | -0.58 |  |  |  |  |  |  |
|  |  |  |  |  |  |  | 0.030 | 0.029 |  |  |  |  |  |  |
|  | | | | | | | | | | | | | | |
| **GNG5** |  |  |  |  |  |  |  |  |  |  |  |  |  | 0.56 |
|  |  |  |  |  |  |  |  |  |  |  |  |  |  | 0.036 |
| BMI= Body mass index; SBP=Systolic blood pressure; GLUC=Glucose; TG=Triglyceride; GGT= Gamma-glutamyl transferase; ALB=Albumin; TP=Total protein, Adipo=Adiponectin; CRP=C-reactive protein; IL-6=Interleukin-6; HCY=Homocysteine; BDNF=Brain-derived neurotrophic factor | | | | | | | | | | | | | | |
